# High-Fat Nutritional Challenge Reshapes Circadian Signatures in Murine Extraorbital Lacrimal Glands

**DOI:** 10.1167/iovs.63.5.23

**Published:** 2022-05-19

**Authors:** Sen Zou, Xinwei Jiao, Jiangman Liu, Di Qi, Xiaoting Pei, Dingli Lu, Shenzhen Huang, Zhijie Li

**Affiliations:** 1 Henan Eye Institute, Henan Eye Hospital and Henan Key Laboratory of Ophthalmology and Visual Science, Henan Provincial People's Hospital, People's Hospital of Zhengzhou University, People's Hospital of Henan University, Zhengzhou City, China

**Keywords:** Circadian rhythm, high-fat diet, inflammation, lacrimal glands, metabolic dysfunction, RNA-Seq, secretory response, transcriptome

## Abstract

**Purpose:**

A high-fat diet (HFD) increases the risk of developing many systemic diseases; however, the effects of high fat intake on lacrimal gland functions and the molecular mechanisms underlying these effects are unknown. We explored the effects of an HFD on the circadian rhythms of the extraorbital lacrimal glands (ELGs).

**Methods:**

Male C57BL/6J mice maintained on a 12/12-hour light/dark cycle were fed an ad libitum HFD or normal chow (NC) for 2 weeks. The ELGs were collected from euthanized animals every 3 hours throughout the circadian cycle (24 hours). Using high-throughput RNA-sequencing (RNA-Seq), we studied the circadian transcriptomic profile of the ELGs. Circadian oscillations in cell size, secretion response, lipid deposition, and immune cell trafficking of the ELGs were also analyzed.

**Results:**

An HFD modulated the circadian transcriptomic profile of the ELGs, including the composition, phase, and amplitude of cyclical transcript oscillations, and affected the associated signaling pathways at spatiotemporal levels. HFD feeding significantly altered the normal rhythmic oscillations of ELG cell size, immune cell trafficking, secretion response, and lipid deposition. After dietary reversal in HFD-fed animals, the activity, core temperature, and lipid accumulation in lacrimal glands recovered partially to the level of NC-fed animals. However, the average cell size of the ELGs, the recruitment of immune cells, and the rhythm of lacrimal secretion did not return to the levels of the NC-fed group.

**Conclusions:**

HFD perturbation interferes with the cyclical transcriptomic profile, cell size, immune cell trafficking, and secretion function of the ELGs with a strikingly high sensitivity.

With accelerated global industrialization, a typical Western diet—characterized by high fat (including saturated and trans fats), protein, and sugar contents and significantly reduced intakes of fruits, vegetables, and fibers^1^^–^^3^—has been adopted globally. Compared to traditional dietary lifestyles, the Western diet significantly increases the risk of obesity,^4^ diabetes,^5^^,^^6^ hypertension,^7^ non-alcoholic fatty liver disease,^8^^,^^9^ metabolic syndrome,^10^ and cardiovascular disease.^11^ With the increase in human life expectancy, the incidence of these diseases has concomitantly increased, resulting in significant healthcare costs.^12^^,^^13^

The effects of the Western diet on the visual system have been studied in recent years.^14^ A high-fat diet (HFD) increases the susceptibility and incidence of age-related eye diseases such as macular degeneration and retinal dysfunction.^15^^–^^18^ Recent studies have demonstrated that HFD-fed mice showed various pathological alterations in retinal structure.^19^^,^^20^ Mice fed an HFD for 10 days exhibited corneal dysfunction, including diminished corneal sensitivity and circadian activities (limbal neutrophil accumulation and epithelial cell division).^21^ Following corneal abrasion, these mice showed delayed wound closure and nerve recovery and dysregulated leukocyte recruitment to the abraded cornea.^21^ Similarly, mice fed an HFD for 10 weeks had hypertrophic meibomian glands and increased levels of lipid species in the meibum.^22^ HFD feeding also leads to decreased^22^^,^^23^ or increased tear production (∼50%) and induces dry eye-like ocular surface damage in murine models.^24^^,^^25^ However, the effects of high fat intake on normal ocular surface homeostasis and disease in humans are not fully appreciated. As humans evolved, the ratio between the two essential fatty acid groups in the diet, the omega-6 (n-6) and omega-3 (n-3) families, was approximately 1:1.^26^ Recently, several clinical and epidemiological studies have shown a correlation between the n-6:n-3 ratio in the diet and the risk of occurrence of dry eye, especially in the postmenopausal female population.^27^^–^^29^ For example, when the ratio of n-6 to n-3 exceeds 15:1, the incidence of dry eye disease may increase 2.5-fold due to the pro-inflammatory properties of omega-6; in contrast, when the amount of omega-3 in the diet is increased, the risk of dry eye may be reduced through the anti-inflammatory effects of n-3.^30^^–^^33^ Unfortunately, the current Western diet tends to be deficient in n-3 fatty acids, with the n-6:n-3 ratio approaching 17:1, depending on geographic location.^34^ Despite these important discoveries, the molecular mechanisms of how nutritional challenges from a Western diet affect the lacrimal glands are poorly studied.

Circadian rhythms are biochemical oscillations that regulate organismal physiology, metabolism, and behavior within the roughly 24-hour cycle.^35^ In mammals, the entrainment of circadian rhythms depends mainly on the melanopsin cells in the retina, which sense external light and transmit it to the suprachiasmatic nucleus (SCN) in the hypothalamus.^36^ As a central master clock, the SCN entrains and synchronizes the peripheral circadian clock (present in almost all mammalian tissues) via neural and endocrine pathways, thereby coordinating the diurnal rhythms in behavior and physiology.^37^ When the circadian rhythm is interrupted due to various stimuli, many physiological processes are altered, leading to many diseases such as sleep disorder, obesity, type 2 diabetes, and cancers.^38^ Although light is the dominant entrainment signal for the master clock, recent studies have shown that some non-SCN pathways—such as food timing and environmental temperature—can also entrain the circadian physiology as the principal zeitgeber (time cues) for the SCN.^39^^–^^42^ Among these non-SCN pathways, nutrient changes in food composition significantly affect the circadian behaviors and physiological functions of humans and rodents.^41^^,^^43^ Studies in this field have mainly focused on the metabolic system,^44^^,^^45^ central nervous system,^46^ and immune system.^13^^,^^47^ However, the effects of HFD feeding on the circadian rhythmicity of the lacrimal gland and the underlying mechanisms of these effects are poorly understood.

The lacrimal gland supports the eye by maintaining ocular surface stability and corneal optic function through the secretion of the aqueous layer of the tear film.^48^^–^^50^ However, tear secretion is affected by many factors, including sex hormones and aging.^51^^,^^52^ Similar to other secretory glands, the secretory function and characteristics of the lacrimal gland are closely regulated by circadian rhythms. The secretion amount,^53^ pH value,^54^ osmotic pressure,^55^ and protein content of the tear film^56^ show a robust rhythmic change in a 24-hour cycle. A high-fructose intake (10 days) can reprogram the circadian clock of murine extraorbital lacrimal glands (ELGs) at the transcriptomic level, especially metabolic- and immunity-associated transcription and some signaling pathways.^57^ Moreover, streptozotocin-induced hyperglycemia dramatically alters the circadian functioning of the ELGs both at the transcriptomic level and in terms of cell size, immune cell trafficking, and secretion response.^58^ Thus, the clock system of ELGs is highly plastic and responds to nutritional challenges; however, little is known about how HFD nutritional challenges affect the clock of the lacrimal gland.

Given the above knowledge, we hypothesized that HFD feeding alters the physiological processes of lacrimal glands underlying their circadian rhythm. To elucidate this, we analyzed the circadian transcriptomic profiles and integrated data for cell size, leukocyte trafficking, and tear secretory response of murine ELGs after HFD treatment (2 weeks). The results demonstrated that even short-term HFD can significantly compromise the circadian rhythmicity of the lacrimal gland, even to the extent of altering its structure and secretory function, and augment the increased tendency to develop dry eye disease. Therefore, this study emphasized the importance of diet composition and a balanced diet to maintain the structure and function of the lacrimal glands. Furthermore, when balanced dietary patterns are restored, alterations in the lacrimal gland, particularly immune cell trafficking and tear secretion, do not fully return to normal levels in the short term, emphasizing the poor reversibility of lacrimal gland damage caused by HFDs. Collectively, our findings shed light on the molecular mechanisms underlying the pathophysiological damage to the lacrimal glands induced by changes in dietary nutrients.

## Materials and Methods

### Animals and Dietary Intervention

All animal experiments were performed in accordance with the guidelines of the Animal Ethics Committee of Henan Provincial People's Hospital and the guidelines in the ARVO Statement for the Use of Animals in Vision and Ophthalmic Research. Eight-week-old male C57BL/6J mice were purchased from the Model Animal Research Center of Nanjing University, and CX3CR1^GFP^ mice were obtained from The Jackson Laboratory (Bar Harbor, ME, USA). All mice were housed in specific pathogen-free barrier facilities and fed in temperature- and humidity-controlled and well-ventilated circadian rhythm cabinets (12/12-hour light/dark daily cycle; Longer-Biotech Co., Ltd., Guangzhou, China).^40^ Zeitgeber time (ZT) was used to record the time: ZT0 for lights on (7 AM) and ZT12 for lights down (7 PM).^58^ All mice were provided with ad libitum access to their respective diets throughout the feeding period.

**Figure 1.**
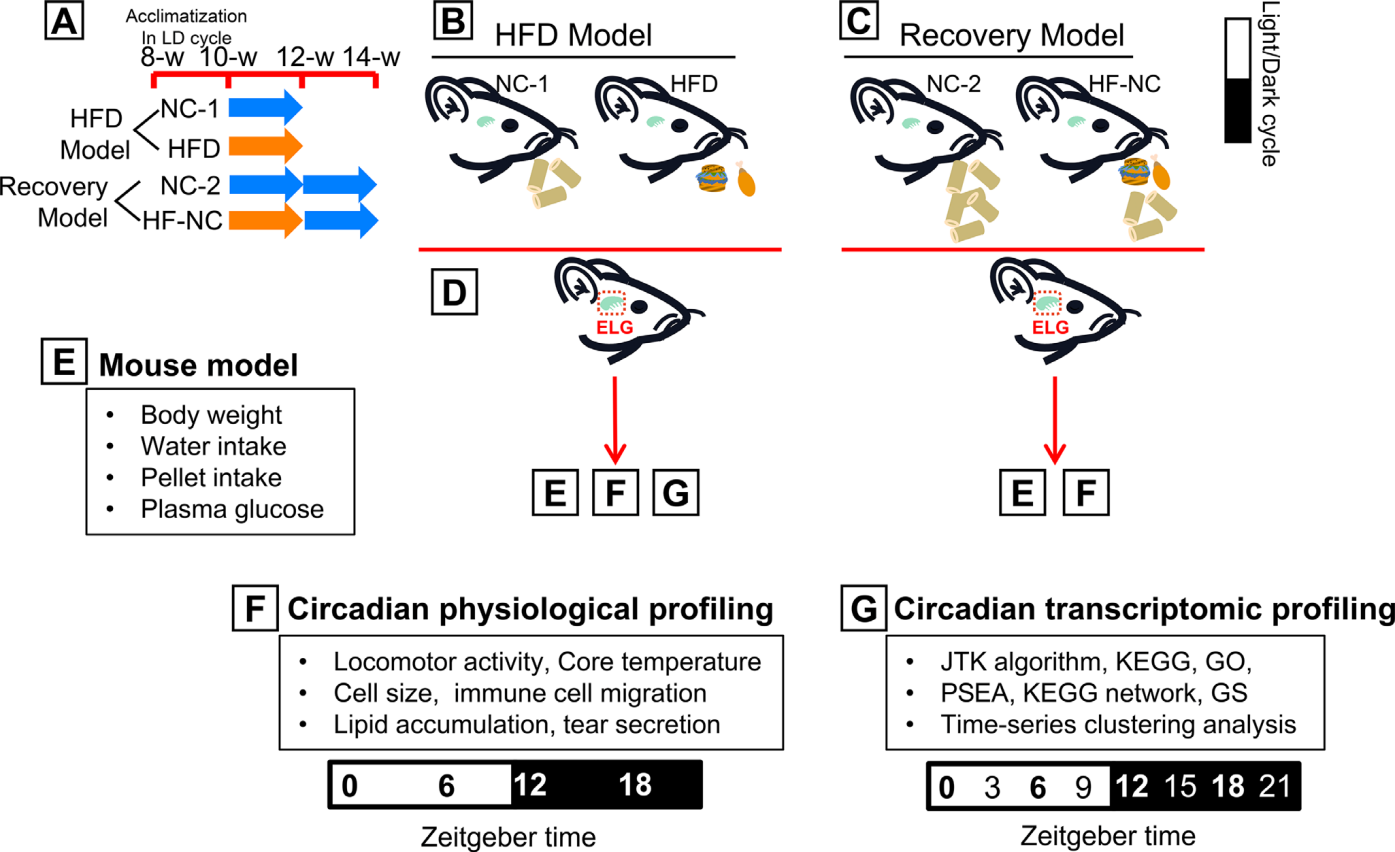
Experimental design and analytical flow. (**A**–**C**) After adapting to the 12-hour light/12-hour dark cycle for 2 weeks, the animals were randomly divided into four groups. The dietary intervention methods were as follows: HFD model (NC-1, normal chow for 2 weeks; HFD, high-fat diet for 2 weeks) and recovery model (NC-2, normal chow for 4 weeks; HF-NC, HFD for 2 weeks and then switched to NC diet for another 2 weeks). (**D**) ELGs were collected from euthanized mice every 3 hours (for HFD model) or 6 hours (for recovery model) over the circadian cycle. (**E**) The mouse model was constructed, and the data of body weight, plasma glucose, water, and food intake of the mice were monitored during the dietary intervention. (**F**) The circadian physiological profiling was comprised of the behavioral rhythm of mice (including analysis of locomotor activity and core temperature oscillations) and phenotypic profiling of ELGs (circadian change in cell size, tear secretions, lipid accumulation, and immune cell trafficking of the ELGs). (**G**) High-throughput RNA-sequencing (RNA-Seq) was performed after removal of the mouse lacrimal glands at the indicated 3-hour intervals over eight time points. Circadian transcripts were selected from RNA-Seq data by the JTK_CYCLE algorithm, and they were then analyzed for alterations in phase, pathways, transcriptomic map, biological processes, and molecular functions using KEGG, GO, PSEA, GSEA, and time-series clustering analysis.

After 2 weeks of adaption in the circadian rhythm cabinets, the mice (10 weeks old) were randomized into four groups. The normal chow 1 (NC-1) group was fed with normal chow (9% kcal fat; Trophic Animal Feed High-Tech Co., Ltd., Nantong, China) for 2 weeks (Supplementary Table S1). The HFD group was fed with an HFD (60% kcal fat; Trophic Animal Feed High-Tech Co., Ltd.) for 2 weeks (Supplementary Table S1). The normal chow 2 (NC-2) group was fed with normal chow (9% kcal fat; Trophic Animal Feed High-Tech Co., Ltd.) for 4 weeks. Finally, the HF-NC group (i.e., the diet reversal group) was fed an HFD for 2 weeks and then switched to an NC diet for another 2 weeks (Figs. 1A–1C). Water intake, food intake, and body weight were measured at the same zeitgeber time every 2 days in 10- to 12-week-old mice in the NC-1 and HFD groups, and in 10- to 14-week-old mice in the NC-2 and HFD-NC groups.

The following data were collected at different ZTs (Fig. 1D). First, general physiological observations, including animal weight, food intake, water intake, and plasma glucose, were recorded (Fig. 1E). Blood glucose concentrations were measured with an Accu-Chek Active glucometer (Roche, Basel, Switzerland) using blood samples collected from the tail vein at the same ZT after fasting for 10 hours at 10, 12, and 14 weeks of age. None of the animals met the diabetes criterion (fasting blood glucose levels > 11.1 mmol/L)^59^^,^^60^ throughout the study period. Second, circadian behavioral observations (i.e., animal locomotor activity and core body temperature) were recorded using a PDT 4000 HR E-Mitter transponder (Mini Mitter, Bend, OR, USA) implanted in the peritoneal cavity (detailed in the General Behavioral Analysis section) (Fig. 1F). Third, circadian changes in cell size, tear secretions (see the Measurement of Aqueous Tear Secretion Response section), lipid accumulation, and immune cell trafficking (see the Immunohistochemistry of ELGs section) of the ELGs were documented (Fig. 1F). Fourth, transcriptomic data for circadian analysis were acquired (see the ELGs Collection and Total RNA Extraction section). Circadian transcripts were selected from RNA-sequencing (RNA-Seq) data by the Jonckheere–Terpstra–Kendall (JTK)_CYCLE algorithm, and were used to analyze alterations in phase, amplitude, signaling pathways, biological processes, and molecular function annotations using Kyoto Encyclopedia of Genes and Genomes (KEGG) analysis, Phase Set Enhanced Analysis (PSEA), Gene Set Enriched Analysis (GSEA), time-series clustering analysis, and Gene Ontology (GO) analysis after different dietary interventions (Fig. 1G). The animals used in this study were euthanized by cervical dislocation after inhalation of ether.

### General Behavioral Analysis

After exposure to different dietary regimens, we measured the locomotor activity (5 minutes for each interval) and core body temperature (20 minutes for each interval) of individually housed mice using the Mini Mitter telemetry system, including 10- to 12-week-old mice (NC-1 and HFD groups) and 10- to 14-week-old mice (NC-2 and HFD-NC groups), as described previously.^58^^,^^61^ Although anesthetized by an intraperitoneal injection of pentobarbital sodium (80 mg/kg of body weight; Sigma-Aldrich, St. Louis, MO, USA), the animals were implanted with a PDT 4000 HR E-Mitter in the peritoneal cavity. The mice were allowed to recover for 1 week before dietary intervention.

### Measurement of Aqueous Tear Secretion Response

After dietary intervention, the lacrimal gland secretion response was measured at ZT0, ZT6, ZT12, and ZT18 at 12 weeks (NC-1 and HFD groups) and 14 weeks (NC-2 and HFD-NC groups), as described previously.^58^^,^^62^ Briefly, pilocarpine hydrochloride was intraperitoneally injected (4.5 mg/kg in saline). After a rest time of 10 minutes, one end of a phenol red–soaked thread was lightly placed on the medial canthus for 20 seconds, and the length of red color on the thread was measured. To observe the effect of HFD and HF-NC treatment, tear secretion response was measured within one 24-hour cycle at 6-hour intervals after the end of the dietary intervention.

### Immunohistochemistry of ELGs

After exposure to different dietary regimens, ELGs were collected from euthanized mice at 6-hour intervals over the circadian cycle (ZT0, ZT6, ZT12, and ZT18) at 12 weeks (NC-1 and HFD groups) and 14 weeks (NC-2 and HFD-NC groups). Histological evaluation of the ELGs was performed using immunohistochemistry as described previously.^63^ Sections were incubated with anti-β-catenin antibody (GB11015; Servicebio Company, Wuhan, China) to identify cell borders, anti-Ly6g antibody (GB11229; Servicebio Company) for neutrophils, anti-CD4 antibody (GB13064-2; Servicebio Company) for CD4^+^ T lymphocytes, and anti-CD8 antibody (GB13429; Servicebio Company) for CD8^+^ T lymphocytes. The cell nuclei of murine ELGs were stained with Mayer's hematoxylin solution (G1004; Servicebio Company). To stain CX3CR1^+^ macrophages, frozen lacrimal gland sections were prepared from CX3CR1^GFP^ mice, and the nuclei were labeled with 4′,6-diamidino-2-phenylindole (G1012-10ML; Servicebio Company). The sectioned lacrimal glands were photographed by light microscopy (Pannoramic 250 MIDI; 3DHISTECH Ltd., Budapest, Hungary). The average cell size of lacrimal glands was calculated from the ratio of the cell number to field size. To quantify the mean number of different leukocyte subsets over a 24-hour period, Ly6g^+^ neutrophils, CD4^+^ T lymphocytes, CD8^+^ T lymphocytes, and CX3CR1^+^ macrophages were counted, and the percentage of each leukocyte subset was calculated as the ratio of the number of positive cells to the total number of cells per unit square. To exclude the presence of non-specific staining, isotype antibody (rabbit IgG, GB111738; Servicebio Company) instead of primary antibody was used as negative control. For lipid staining in ELGs, samples frozen in liquid nitrogen were stained with Oil Red O (ORO, G1016; Servicebio Company) as previously described.^63^

### ELGs Collection and Total RNA Extraction

After dietary intervention, ELGs were collected from euthanized mice every 3 hours for eight time points over the circadian cycle (ZT0, ZT3, ZT6, ZT9, ZT12, ZT15, ZT18, and ZT21) at 12 weeks for the NC-1 and HFD groups, as previously described.^58^^,^^63^ Briefly, total RNA was isolated from the ELGs of NC-1 and HFD animals collected at the different ZTs using the RNeasy Spin Column Kit (QIAGEN, Hilden, Germany) according to the manufacturer's instructions. At each time point, RNA-Seq analysis was performed using three biological replicates. In addition, tissue samples that were collected during the dark phase, when the animals were kept in the dark, were collected under dim red illumination (3-watt safe lamp, DG-20A; Jining Hengshuo Testing Instrument Co., Ltd., Shandong, China), as previously described.^64^

### Library Preparation and RNA-Seq

Library preparation and sequencing were performed as previously described.^57^^,^^58^ After total RNA was qualified and quantified using a NanoDrop spectrophotometer (Thermo Fisher Scientific, Waltham, MA, USA) and Agilent 2100 bioanalyzer (Agilent Technologies, Santa Clara, CA, USA), mRNA was purified using Dinabeads Oligo(dT)_25_ (Thermo Fisher Scientific) magnetic beads. Purified mRNA was fragmented into small pieces with fragment buffer. First-strand cDNA was then generated by random hexamer-primed reverse transcription, followed by second-strand cDNA synthesis. Afterward, end repair was performed by incubation with the addition of A-Tail mix and RNA index adapters. The cDNA fragments were amplified by polymerase chain reaction (PCR), and products were purified using AMPure XP beads (Beckman Coulter, Brea, CA, USA). The product was verified on an Agilent Technologies 2100 Bioanalyzer for quality control. The double-stranded PCR products were denatured by heating and cyclized using the splinted ligation method. The final single-stranded library was amplified by circular replication using phi29 (Thermo Fisher Scientific) to make DNA nanoballs, where one molecule had more than 300 copies. The DNA nanoballs were then loaded into patterned nanoarrays to generate single-end 50-base (SE50) reads on the BGISEQ500 platform (BGI, Guangdong, China) with a read depth of 20 million.

Subsequently, the raw reads obtained from sequencing were filtered using SOAPnuke v1.5.2, developed by BGI independently for sequencing data filtering.^65^ HISAT2 v2.0.4^66^ and Bowtie2 v2.2.5 were used to align the clean reads to the reference gene sequence (species, *Mus* *musculus*; source, National Center for Biotechnology Information [NCBI]; version, GCF_000001635.26_GRCm38. p6),^67^ and then RSEM v1.2.8 was used to calculate the gene expression level of each sample.^68^ The average alignment rate of the sample alignment genome and gene set were all over 90%. The RNA integrity number (RIN) scores were in the range of 8.1 to 9.0 (28S/18S, 1.1–1.4), and the baseline was flat, indicating that the RNA integrity met the requirements for subsequent sequencing.

### Analysis of Rhythmic Genes

To evaluate the circadian transcriptomes of ELGs over a 24-hour cycle, the JTK_CYCLE algorithm (https://openwetware.org/wiki/File:JTKversion3.zip) was used with R (R Foundation for Statistical Computing, Vienna, Austria) to determine genes with rhythmic expression.^58^ Time-ordered fragments per kilobase of transcript per million mapped fragments (FPKM) of transcribed genes were input in the JTK_CYCLE algorithm, and rhythmic genes with an oscillation period of 24 hours were screened out. Thus, all genes were divided into three categories: (1) low expression genes, genes with FPKM < 0.1; (2) rhythmic genes, genes with FPKM ≥ 0.1 and Bonferroni-adjusted *P* < 0.05 (JTK results); and (3) non-rhythmic genes, genes with FPKM ≥ 0.1 and Bonferroni-adjusted *P* ≥ 0.05. The phase and amplitude of the rhythmic genes were also measured by the algorithm.

### Functional Annotation with KEGG, GO, PSEA and GSEA

To elucidate the relationship between ELG transcripts and potential biological functions, KEGG annotation was performed using BlastAll software (NCBI, Bethesda, MD, USA) with the KEGG database and NCBI RefSeq as the reference gene set.^57^ The resulting annotations were grouped into clusters according to the common gene members. The significance threshold of the *Q* value (*P* value adjusted by the Benjamini and Hochberg [BH] method) was set at 0.05. The KEGG network diagram was used to illustrate the relationships between genes and the enriched KEGG pathways.

GO analysis was used to analyze the molecular functions and biological processes of genes. Candidate genes were mapped to each term in the GO database (http://www.geneontology.org), and the number of genes in each term was calculated. The significance threshold of the *Q* value (adjusted *P* value by BH) was set at 0.05.

To annotate the function of rhythmic genes at the level of oscillating phases, PSEA v1.1 software was used along with the annotated reference gene set (c2.cp.kegg.v7.2.symbols.gmt) from the Molecular Signatures Database (MSigDB; https://www.gsea-msigdb.org/gsea/msigdb/).^58^ The domain circadian phase was from 0 to 24, and the items per set was set at 10 to 1000 with a *Q* value (determined by Kuiper test) < 0.05.

GSEA v3.0 software (Broad Institute of MIT and Harvard, Cambridge, MA, USA) was used to characterize biological pathways. The annotated reference gene sets, c5.go.bp.v7.2.symbols.gmt and c2.cp.kegg.v7.2.symbols.gmt, were downloaded from the MSigDB. The pathways with nominal *P* < 0.05 and a *Q* value (adjusted by BH) < 0.25 were considered significantly enriched.

### Time-Series Clustering Analysis

To more intuitively show the dynamic trends of rhythmic gene expression levels in the ELGs over time, we performed noise-robust soft clustering analysis using the fuzzy c-means clustering algorithm in the Mfuzz package, as previously described (http://www.bioconductor.org/packages/release/bioc/html/Mfuzz.html).^58^ The parameters were as follows: core threshold, 0.7; number of clusters, 4; and default values for the other parameters.

### Statistical Analysis and Software

Prism 8.3.0 (GraphPad, La Jolla, CA, USA) was used for statistical analysis and figure preparation. The heat map was generated using the phenomap package in R (64-bit, version 3.6.1). The Venny diagram plotter (Venny 2.1.0, http://bioinfogp.cnb.csic.es/tools/venny/index.html) was used to analyze the number of overlapping genes in different gene sets. Oriana 4.01 (Kovach Computing Services, Pentraeth, Wales, UK) was used to illustrate the phase, period distribution, and Rayleigh vector of the rhythmic genes. Data were tested for normal distribution before all statistical analyses. A Student's *t*-test or one-way ANOVA with Bonferroni correction was used for statistical analysis. A nonparametric Mann–Whitney test was used to compare the differences between groups of data with non-normal distributions. *P* < 0.05 indicated a statistically significant difference.

## Results

### HFD Alters Animal General and Circadian Behavior

**Figure 2.**
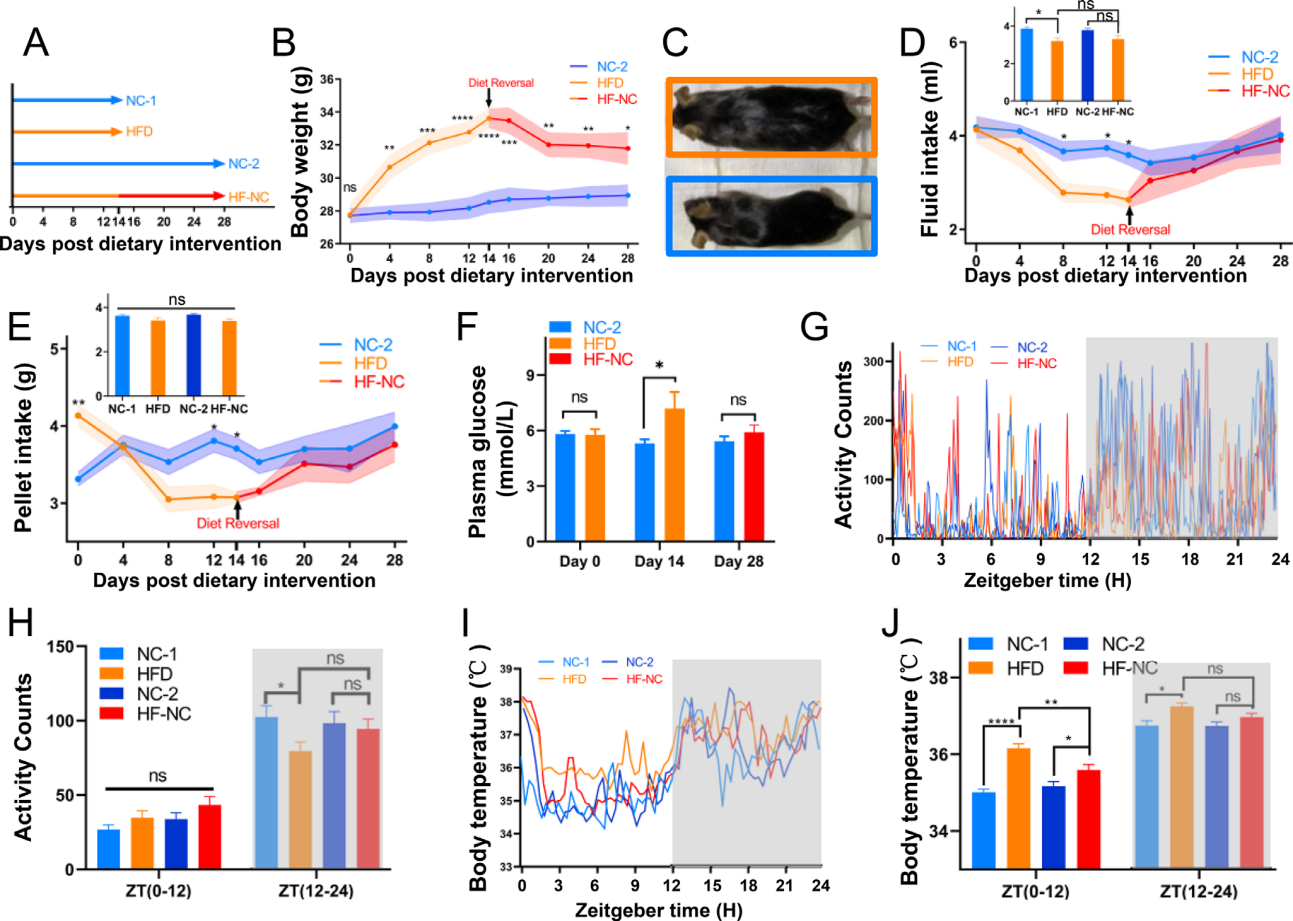
HFD feeding increases body weight and affects animal behavior. (**A**) The 10-week-old male mice were randomly divided into four groups with different dietary schemes: NC-1, normal chow for 2 weeks; HFD, high-fat diet for 2 weeks; NC-2, normal chow for 4 weeks; HF-NC, HFD for 2 weeks and then switched to NC diet for another 2 weeks. (**B**) Age-matched male C57BL/6J mice were placed on NC or HFD feeding. The diet reversal (HF-NC) mice were fed an HFD for 2 weeks and then switched to an NC for another 2 weeks. Body weight was measured every 2 days; *n* = 12 mice per group. (**C**) Body size was higher in the HFD mice than in the NC mice at day 14. (**D**) Water intake in mice during 28 days after the start of the feeding experiment. The *top left inset* depicts the average daily water intake of each mouse; *n* = 12 mice per group. (**E**) Food intake in mice for 28 days after the start of the feeding experiment. The *top left inset* depicts the average daily pellet intake of each mouse; *n* = 12 mice per group. (**F**) Plasma glucose concentration in fasting animals; *n* = 6 mice per group. (**G**) The representatively rhythmic characteristics of locomotor activity in animals in all four groups over the last 24-hour light cycle (12 hours light/12 hours dark) of the dietary intervention. *Gray shading* indicates dark phase. (**H**) Comparisons of locomotor activity among animal groups over the last 24-hour light cycle (12 hours light/12 hours dark) during the dietary intervention; *n* = 6 mice per group. *Gray shading* indicates dark phase. (**I**) The representatively rhythmic characteristics of core body temperature in animals in the four groups over the last 24-hour light cycle (12 hours light/12 hours dark) of the dietary intervention, respectively. *Gray shading* indicates dark phase. (**J**) Comparisons of core body temperature between animal groups over the last 24-hour light cycle (12 hours light/12 hours dark) during the dietary intervention; *n* = 6 mice per group. *Gray shading* indicates dark phase. *P < 0.05, **P < 0.01, ***P < 0.001, ****P < 0.0001; ns, not significant.

The age and diet intervention strategies of mice are shown in Figure 2A. The weight of the mice gradually increased during the 2 weeks of HFD feeding intervention, but significant differences were observed only from the fourth day after the intervention (Figs. 2B, 2C). The average daily water intake was significantly lower in the HFD-fed group than in the NC-fed group (Fig. 2D), although the average daily food intake was not significantly different (Fig. 2E). The fasting blood glucose concentrations of the HFD-fed mice tended to be higher than those of the NC-fed mice (Fig. 2F). In NC-fed mice, locomotor activity and core body temperatures from ZT12 to ZT24 were higher than those from ZT0 to ZT12 (Figs. 2G–2J). Consistent with the results of previous studies,^40^^,^^69^ HFD feeding for 2 weeks did not affect the locomotor activity of mice during the daytime (ZT0–ZT12) but significantly reduced the locomotor activity from ZT12 to ZT24 (Figs. 2G, 2H; Supplementary Fig. S1A). The core body temperature of HFD-fed mice was significantly higher than that of NC-fed mice during the dark and light phases (Figs. 2I, 2J; Supplementary Fig. S1C).

Following the diet reversal regimen for 2 weeks, the body weight of HF-NC mice was still significantly higher than that of NC-fed mice (Fig. 2B). However, compared with the NC-fed group, there was no significant difference in fluid consumption, pellet consumption, and fasting blood glucose level in the HF-NC group (Figs. 2D–2F). After 2 weeks of the dietary reversal regimen, the locomotor activity of HFD-fed mice returned to the level of the NC-2 group (Figs. 2G, 2H; Supplementary Fig. S1B). Although the core body temperature of HFD-fed mice decreased from ZT0 to ZT12 after 2 weeks of dietary reversal intervention, it was still significantly higher than that of the NC-2 group (Figs. 2I, 2J; Supplementary Fig. S1D). From ZT12 to ZT24, the core temperature decreased and approached the core temperature of the NC-2 group (Fig. 2J). These results indicate that short-term HFD feeding significantly altered the general and circadian rhythmic behavior of mice. The diet reversal regimen restored most of the normal parameters other than the rhythm of locomotor activities and core body temperature.

### HFD Alters Cycling Alteration of ELGs Cell Size

**Figure 3.**
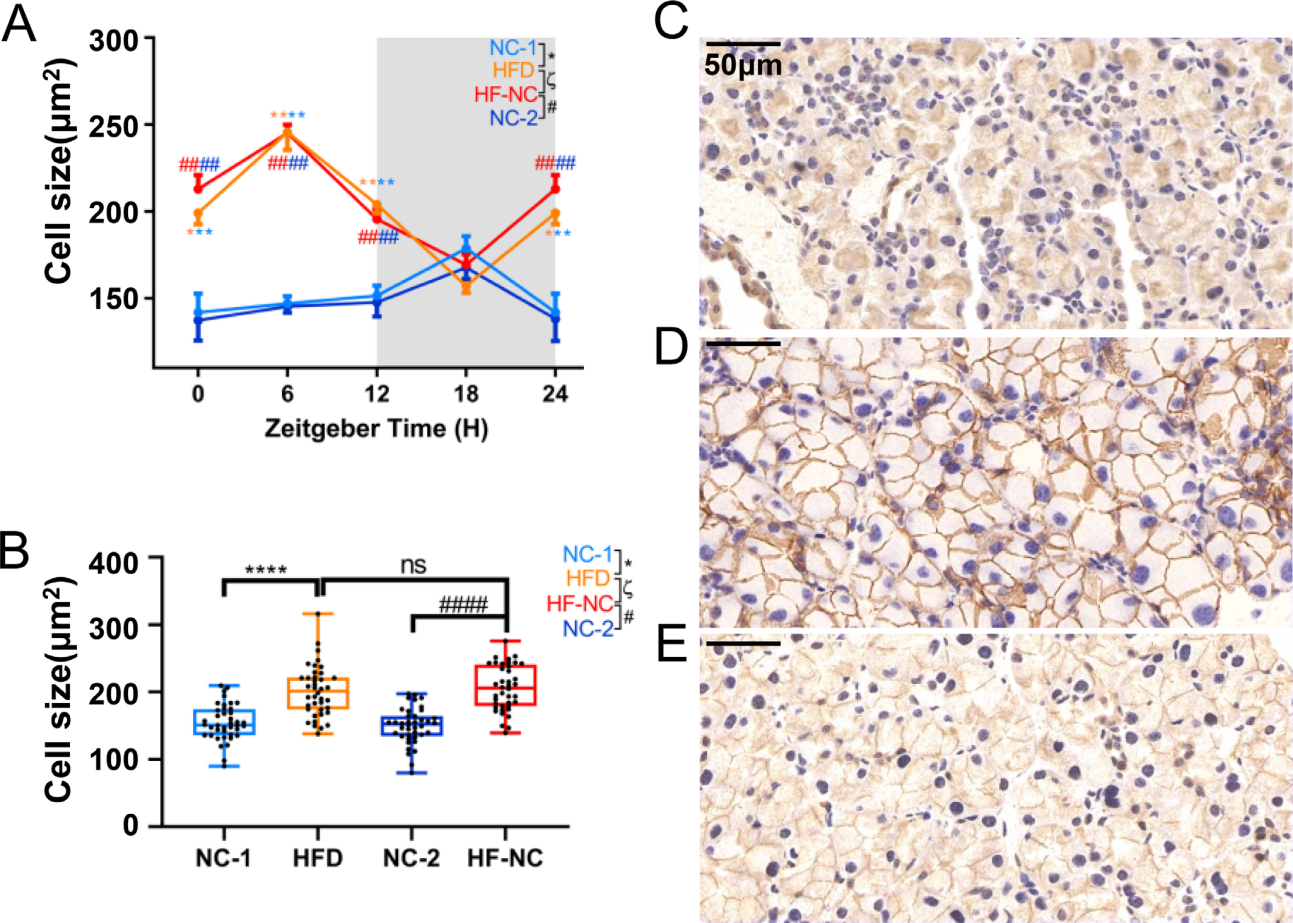
HFD affects cell sizes in lacrimal glands. (**A**) Diurnal oscillations of average lacrimal gland cell size in immunohistochemical images of ELG sections from mice euthanized at 6-hour intervals; *n* = 10 mice per group per time point every 6 hours. One-way ANOVA using Bonferroni correction: ^*^significant difference between NC-1 and HFD groups; ^#^significant difference between NC-2 and HF-NC groups; ^ζ^significant difference between HFD and HF-NC groups. ***P < 0.001, ****P < 0.0001. *Gray shading* indicates dark phase. (**B**) Comparison of the size of mouse lacrimal gland cells among the four groups; *n* = 40 mice per group. One-way ANOVA using Bonferroni correction: ^*^significant difference between NC-1 and HFD groups; ^#^significant difference between NC-2 and HF-NC groups; ^ζ^significant difference between HFD and HF-NC groups. ****P < 0.0001; ns, not significant. (**C**–**E**) Representative immunohistochemical images of ELGs from NC-1 (**C**), HFD (**D**), and HF-NC (**E**) mice at ZT6. Magnification, 400×. *Scale* *bar*: 50 µm.

The cell sizes of ELGs change in a circadian rhythm.^63^ To evaluate the effects of an HFD on the rhythm of cell size changes in mouse ELGs, we compared the dynamics of average cell size in NC- and HFD-fed ELGs over a 24-hour cycle under a microscope. Consistent with our past results,^58^ the average cell size of the lacrimal glands in NC-1 mice peaked at ZT18 and troughed at ZT0 (Fig. 3A). However, compared with the average cell size of NC-1 ELGs, that of HFD ELGs peaked at ZT6 and troughed at approximately ZT18 (Fig. 3A). The average cell size of HFD ELGs at ZT0, ZT6, and ZT12 was significantly higher than that of NC-1 ELGs, respectively. Following food reversal treatment, we also observed the recovery of rhythmic patterns of average cell size changes in the ELGs of HFD-fed mice. The results showed that the rhythm of lacrimal gland cells after the food reversal regimen was still comparable to that of the lacrimal gland cells in HFD-fed mice, and there was no recovery to the level of NC-fed ELGs (Fig. 3A). Similarly, the cumulative values of cell sizes over five time points showed that HFD-fed ELGs had significantly larger cells than NC-fed ELGs, and there was no significant difference between HFD-fed and HF-NC–fed ELGs (Figs. 3B–3E). These data suggest that HFD significantly alters the normal rhythmic changes of average cell sizes in lacrimal glands; in the HFD-fed group, the short-term reversal of dietary regimen could not restore the trend of cell size rhythm to that of the NC-fed group.

### HFD Alters the Characteristics of Circadian Transcriptome in Mouse Lacrimal Gland

**Figure 4.**
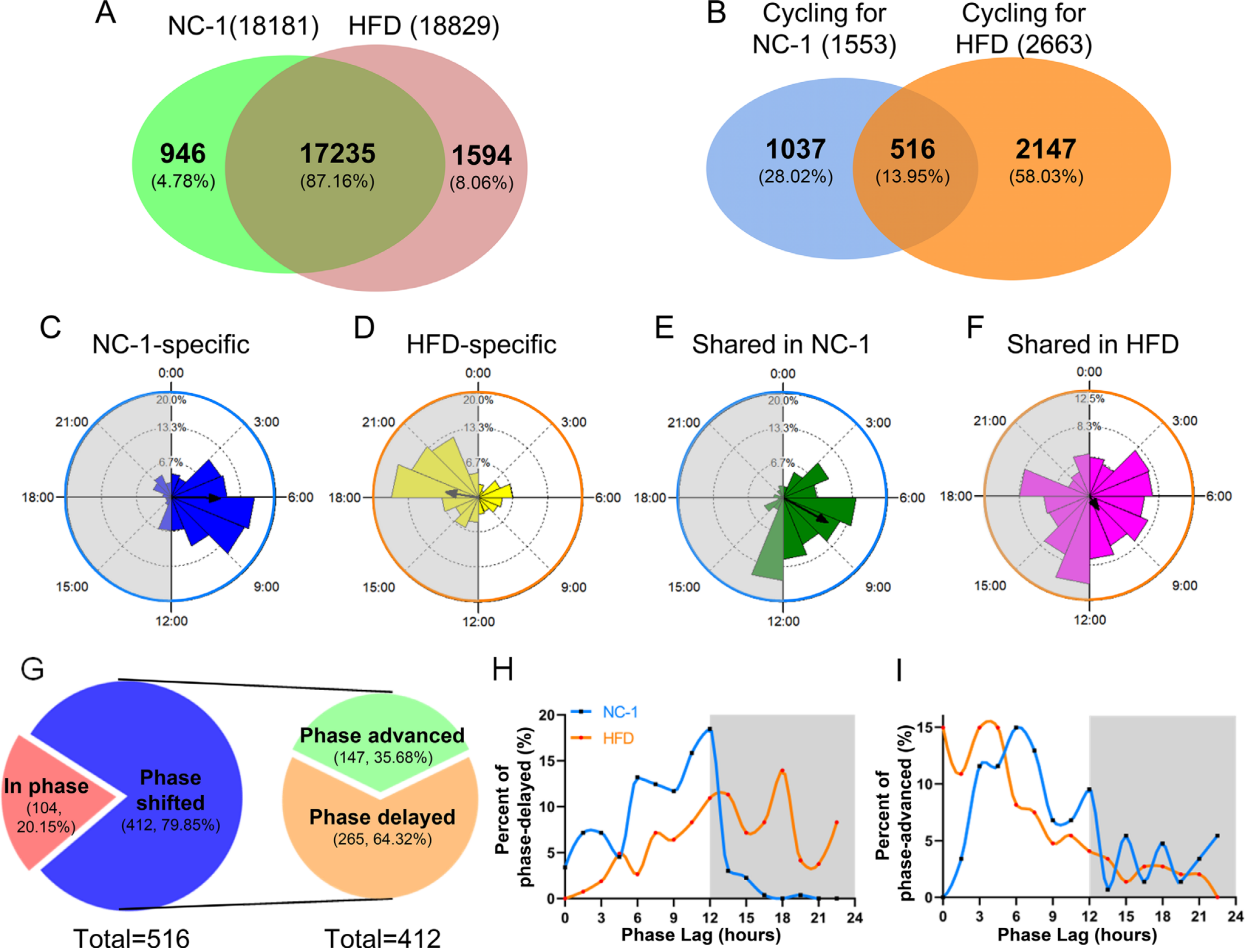
HFD alters the characteristics of the circadian transcriptome in mouse lacrimal glands. (**A**) Venn diagram representing the sets of transcripts from the ELGs of NC-1 and HFD mice; *n* = 3 mice per group per time point every 3 hours (*n* = 24 mice per group). (**B**) Venn diagram exhibiting the similarities and differences in rhythmic transcripts in the ELGs of NC-1 and HFD mice. (**C**–**F**) Phase analysis of oscillating genes in the ELGs. *Gray shading* in the *left panel* indicates the dark cycles. (**C**) NC-1–specific oscillating genes. (**D**) HFD-specific cycling genes. (**E**) Shared oscillating genes in NC-1 mice. (**F**) Shared oscillating genes in HFD mice. (**G**) Phase analysis of shared oscillating genes that follow normal circadian rhythmicity in the ELGs of NC-1 and HFD mice. (**H**, **I**) Phase distribution of phase-delayed (**H**) and phase-advanced genes (**I**) in shared rhythmic transcripts. *Gray shading* indicates dark phase.

To analyze the effects of HFD feeding on circadian transcriptomic profiles in murine ELGs, we investigated the expression of cycling transcripts in ELGs collected at eight time points at 3-hour intervals. We identified 2663 (14.14%) and 1553 (8.54%) circadian transcripts (Supplementary Table S2) from all ELG transcripts of HFD (18,829) and NC-1 (18,181) mice, respectively (Figs. 4A, 4B; Supplementary Table S3). In total, 516 cycling transcripts were shared between the two groups, 1037 were unique to NC-1 ELGs, and 2147 were unique to HFD-fed ELGs (Fig. 4B; Supplementary Table S4). Although the expression of unique transcripts in NC-1 mice peaked during the light phase, they did not display a significant circadian rhythmic pattern in HFD ELGs (Supplementary Figs. S2A, S2C). In contrast, the unique cycling transcripts in HFD mice were mainly expressed during the dark phase but did not show a significant circadian rhythmic expression pattern in NC-1 ELGs (Supplementary Figs. S2B, S2C). To evaluate the temporal effect of an HFD on the circadian transcriptome, we calculated the phase, period, and Rayleigh vector of the cycling transcripts in the shared, NC-1–specific, and HFD-specific cycling transcripts using Oriana software. The results showed that the expression phase of NC-1–specific cycling transcripts was mainly during the daytime (Fig. 4C). The mean vector (µ) was approximately ZT6:07, and its length (*r*) was 0.463 (Fig. 4C). In contrast, the expression phase of HFD-specific cycling transcripts occurred during the dark phase (µ = ZT18:37; *r* = 0.309) (Fig. 4D). Interestingly, the shared cycling transcripts were mainly distributed from ZT3 to ZT13.5 in the NC-1 group (µ = ZT7:59; *r* = 0.484) and throughout the circadian cycle in the HFD group (µ = ZT9:47; *r* = 0.155) (Figs. 4E, 4F). Of the shared transcripts in HFD ELGs, 79.85% were phase shifted and only 20.15% were in phase (Fig. 4G). Of these shifted rhythmic transcripts, 64.32% were delayed in phase, and 35.68% were advanced (Figs. 4G–4I). The amplitude of oscillation in HFD unique cycling transcripts was lower than that in the NC-1 group (Supplementary Fig. S2D, top). Similarly, the amplitude of oscillation of shared transcripts was lower in the HFD group than in the NC-1 group (Supplementary Fig. S2D, bottom). Thus, HFD intervention dramatically altered the composition, number, and oscillation phase of rhythm transcripts in mouse ELGs.

### HFD Alters the Functional Characteristics of Circadian Transcriptomic Genes in Mouse Lacrimal Gland

**Figure 5.**
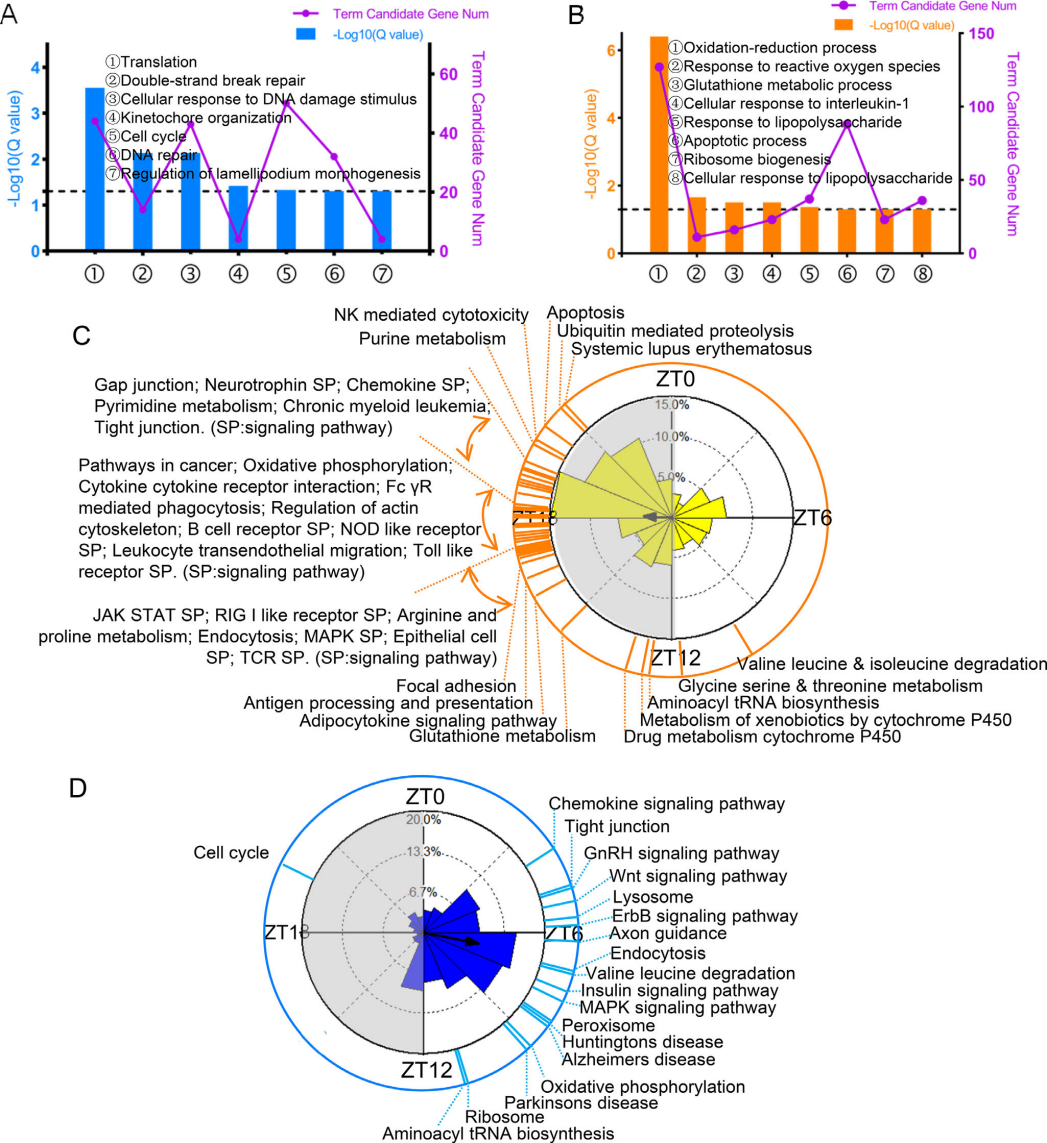
HFD alters the oscillatory characteristics of circadian transcriptomic profiling in mouse lacrimal glands. (**A**) Gene annotation of GO_Biological Process enriched for NC-1–specific rhythmic transcripts (*Q* < 0.05). (**B**) Gene annotation of GO_Biological Process enriched for HFD-specific rhythmic transcripts (*Q* < 0.05). (**C**, **D**) Summary of significantly phase-clustered (*Q* < 0.05) pathways in HFD (**C**) and NC-1 (**D**) ELGs. The inner circle shows the phase distribution of the rhythmic genes. The length of the column represents the number of genes in that phase. The *yellow* or *blue dotted lines* on the outside circle represent the KEGG pathways (*Q* < 0.05 and >10 genes/pathway) enriched at different ZTs based on the phase distribution of the inner circle. *Gray shading* indicates dark phase.

To characterize the effect of an HFD on the potential molecular functions and biological processes of rhythmic genes, we performed GO analysis for NC-fed and HFD-fed ELGs. The NC-1–specific and HFD-specific rhythmic genes were mainly enriched in various biological processes, as shown in Figures 5A and 5B. To evaluate the effect of an HFD on the spatiotemporal distribution of functions of rhythmic genes, we used the PSEA method. The signaling pathways enriched in the NC-1 group were mainly located in the light phase, whereas those in the HFD group were mainly located in the dark phase (Figs. 5C, 5D; Supplementary Tables S5, S6). Importantly, more immune-related pathways were enriched in HFD ELGs (Fig. 5C). Collectively, these results indicated that HFD feeding significantly reshaped the circadian rhythmic activity at both the GO and PSEA levels, which might lead to alterations in the potential functions of these cycling transcripts in HFD-fed ELGs.

### HFD Alters the Cluster-Dependent Transcriptomic Map

**Figure 6.**
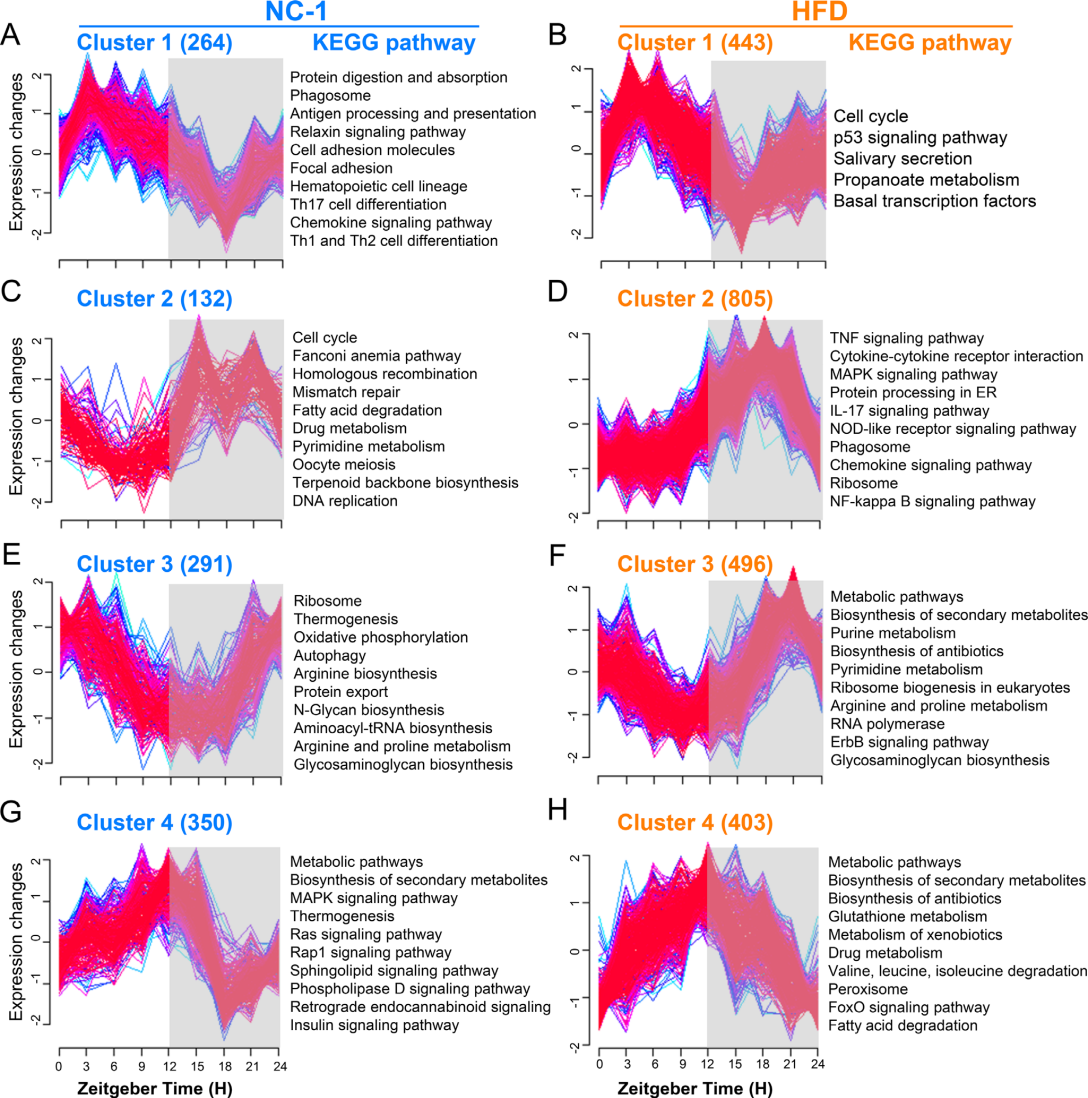
HFD alters the cluster-dependent transcriptomic map. (**A,C,E,G**) Temporal patterns of Z-scores of genes from four enriched clusters for NC-1 ELGs (*left*). Selected enriched KEGG annotations for each cluster are shown (*P* < 0.05) (*right*). (**B**, **D**, **F**, **H**) Temporal patterns of Z-scores of genes from four enriched clusters for HFD ELGs (*left*). Selected enriched KEGG annotations for each cluster are shown (*P* < 0.05) (*right*). Expression values are standardized to have a mean of 0 and a standard deviation of 1. *Blue* and *green lines* represent low-membership-value genes, and *red lines* represent high-membership-value genes. *Gray shading* indicates dark phase.

To visualize the dynamic alterations of rhythmic genes in the transcriptomic profile of ELGs after HFD feeding, the time-series analyses of rhythmic genes were analyzed as previously described.^58^^,^^63^^,^^70^ Based on the positions of the peaks and troughs, four kinds of oscillating clusters were defined, and KEGG analysis was used to annotate the functions of transcripts in each cluster. The results were as follows: In cluster 1, the peaks and troughs were located at ZT3 and ZT18 (Figs. 6A, 6B), and 264 rhythmic genes and 443 rhythmic transcripts were clustered in NC-1 and HFD ELGs, respectively. In cluster 2, the peaks and troughs were located at ZT15 and ZT6 (Figs. 6C, 6D), and 132 and 805 rhythmic transcripts were clustered in NC-1 and HFD ELGs, respectively. In cluster 3, the peaks and troughs were located at ZT0 and ZT12, and 291 and 496 rhythmic transcripts were clustered in NC-1 and HFD ELGs, respectively (Figs. 6E, 6F). In cluster 4, the peaks and troughs were located at ZT12 and ZT21, and 350 and 403 rhythmic transcripts were clustered in NC-1 and HFD ELGs, respectively (Figs. 6G, 6H).

As shown on the right of each panel, the enriched KEGG pathways for transcripts with similar oscillations between NC-1 and HFD ELGs had different annotation functions (Figs. 6A–6H; Supplementary Tables S7, S8). Cluster 1 genes in NC-1 ELGs were related to immune function (Fig. 6A), whereas cluster 2 genes in HFD ELGs were associated with immune function (Fig. 6D). The transcripts of clusters 2 and 3 in NC-1 ELGs were mainly related to metabolic processes (Figs. 6C, 6E), whereas similar pathways in HFD ELGs were concentrated in clusters 3 and 4 (Figs. 6F, 6H). In summary, our results indicate that HFD feeding reprogrammed the clustering patterns and corresponding biological pathways of rhythmic transcripts.

### HFD Alters the Lipid Metabolism in Murine ELGs

**Figure 7.**
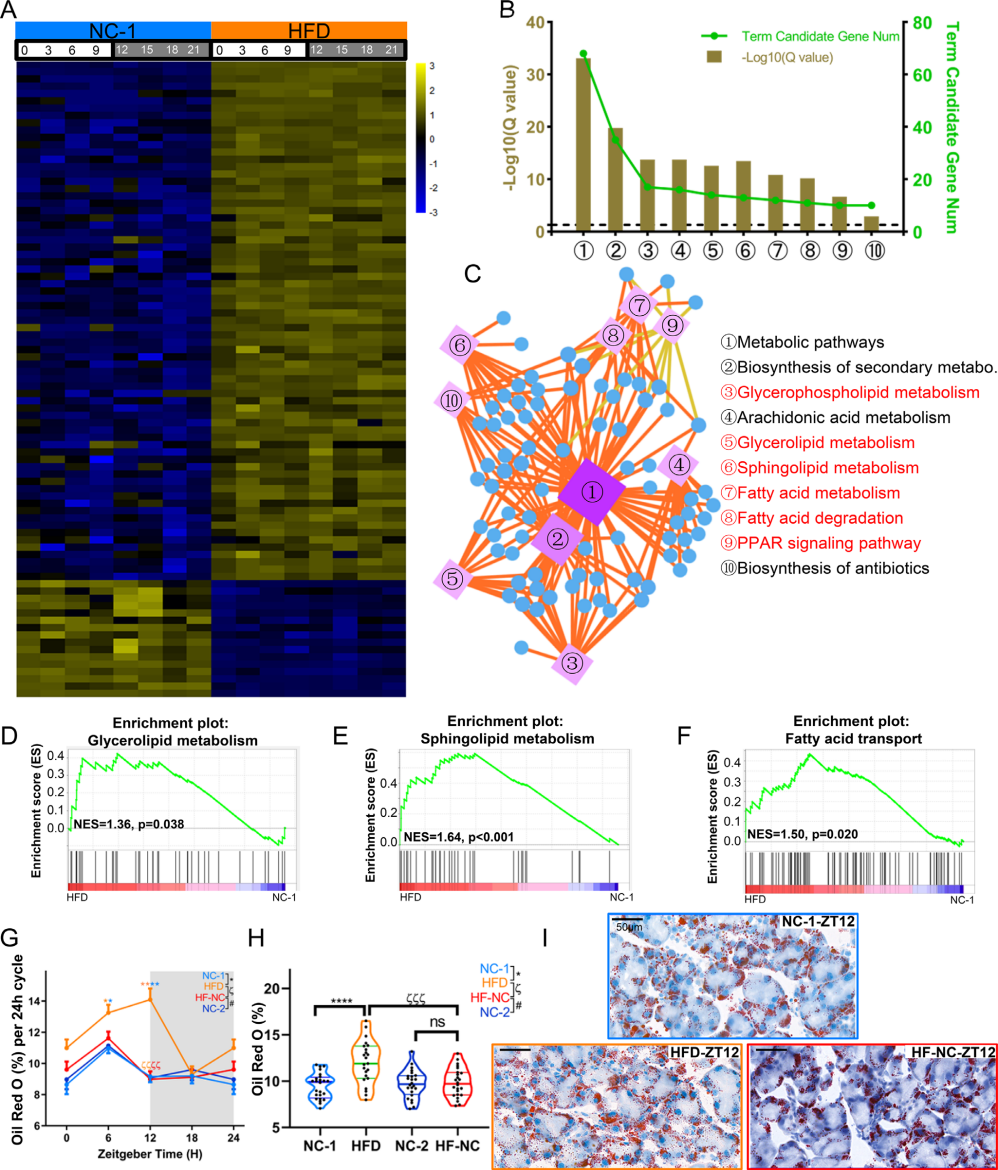
HFD alters the lipid metabolism of murine lacrimal glands at transcriptomic and phenotypic levels. (**A**) The heatmap shows the differential expression levels of lipid metabolism-associated transcripts in the lacrimal glands of NC-1 and HFD mice (fold change ≥ 2 or ≤ 0.5, BH-adjusted *P* < 0.05), based on the RNA-Seq data at eight time points in a 24-hour cycle. The *color bar* indicates the scale for transcript expression from *blue* to *yellow* across eight time points, with the expression range normalized to ±3. (**B**) Annotation of differentially expressed lipid metabolism-associated transcripts from the KEGG database (*Q* < 0.05). The top 10 annotated pathways are shown. The *dashed line* represents *Q* = 0.05. (**C**) KEGG network diagram of differentially expressed lipid metabolism–associated transcripts. The circles represent transcripts. The color and size of the circles indicate the number of transcripts connected to each node. The darker the color and the larger the box, the more transcripts connected to the node. Differently colored lines represent different pathway classifications. The *orange* and *yellow lines* represent metabolic and organismal systems, respectively. (**D**–**F**) GSEA results showing the enrichment plots for glycerolipid metabolism (**D**), sphingolipid metabolism (**E**), and fatty acid transport (**F**) in the ELGs of NC-1 and HFD mice. NES, normalized enrichment score. (**G**) Diurnal oscillations of lipid droplet deposition in the immunohistochemistry images of ELG sections from NC-1, HFD, NC-2, and HF-NC mice at 6-hour intervals; *n* = 6 mice per group per time point (every 6 hours). One-way ANOVA using Bonferroni correction: ^*^significant difference between NC-1 and HFD groups; ^#^significant difference between NC-2 and HF-NC groups; ^ζ^significant difference between HFD and HF-NC groups. **P < 0.01, ****P < 0.0001. *Gray shading* indicates dark phase. (**H**) Quantitative analysis of lipid droplet deposition using one-way ANOVA with Bonferroni correction for multiple testing; *n* = 24 mice per group. One-way ANOVA using Bonferroni correction: ^*^significant difference between NC-1 and HFD groups; ^#^significant difference between NC-2 and HF-NC groups; ^ζ^significant difference between HFD and HF-NC groups. ***P < 0.001, ****P < 0.0001; ns, not significant. (**I**) Lipid droplets visualized by ORO staining in the ELGs of NC-1, HFD, and HF-NC mice at ZT12. Magnification, 400×. *Scale bar*: 50 µm.

To determine the effect of HFD on lipid metabolism-related genes in murine ELG transcriptomes, we screened the differentially expressed transcripts (fold change ≥ 2 or ≤ 0.5, BH-adjusted *P* < 0.05) between the NC-1 and HFD ELGs. In total, we found 87 differentially expressed genes, of which 71 were upregulated and 16 were downregulated (Fig. 7A; Supplementary Table S9). The differentially expressed transcripts were enriched in some signaling pathways (as shown by KEGG analysis; *Q* < 0.05). Figure 7B shows the top 10 pathways. A KEGG network diagram (Fig. 7C; Supplementary Table S10) and GSEA (Figs. 7D–7F) were used to illustrate the aggregations of transcriptional genes in specific functional pathways. The results showed that most significantly enriched genes were closely related to the lipid metabolism pathways.

To verify the effect of HFD on lipid metabolism in ELGs, we used ORO to stain the lipid droplets in mouse ELG tissues collected every 6 hours over a 24-hour cycle. ImageJ (National Institutes of Health, Bethesda, MD, USA) analysis revealed that the content of lipid droplets showed a circadian oscillation in normal lacrimal glands and that lipid accumulation peaked at ZT6 and troughed at ZT0 (Fig. 7G). However, after HFD feeding for 2 weeks, the lipid accumulation peaked at ZT12 and troughed at ZT18 (Fig. 7G). We also analyzed whether the HF-NC diet reversal regime reversed the lipid accumulation in lacrimal glands by examining the alteration of lipid staining in ELG tissues after feeding reversal for 2 weeks. The level of lipid accumulation recovered approximately to the level of the NC-fed ELGs (Figs. 7G–7I). Overall, these data suggest that HFD feeding altered the normal metabolic processes of murine ELGs, especially the lipid-associated signaling pathways, leading to lipid deposition in the ELGs. Diet reversal also reversed the lipid accumulation.

### HFD Alters Immune Homeostasis in Murine Lacrimal Glands

**Figure 8.**
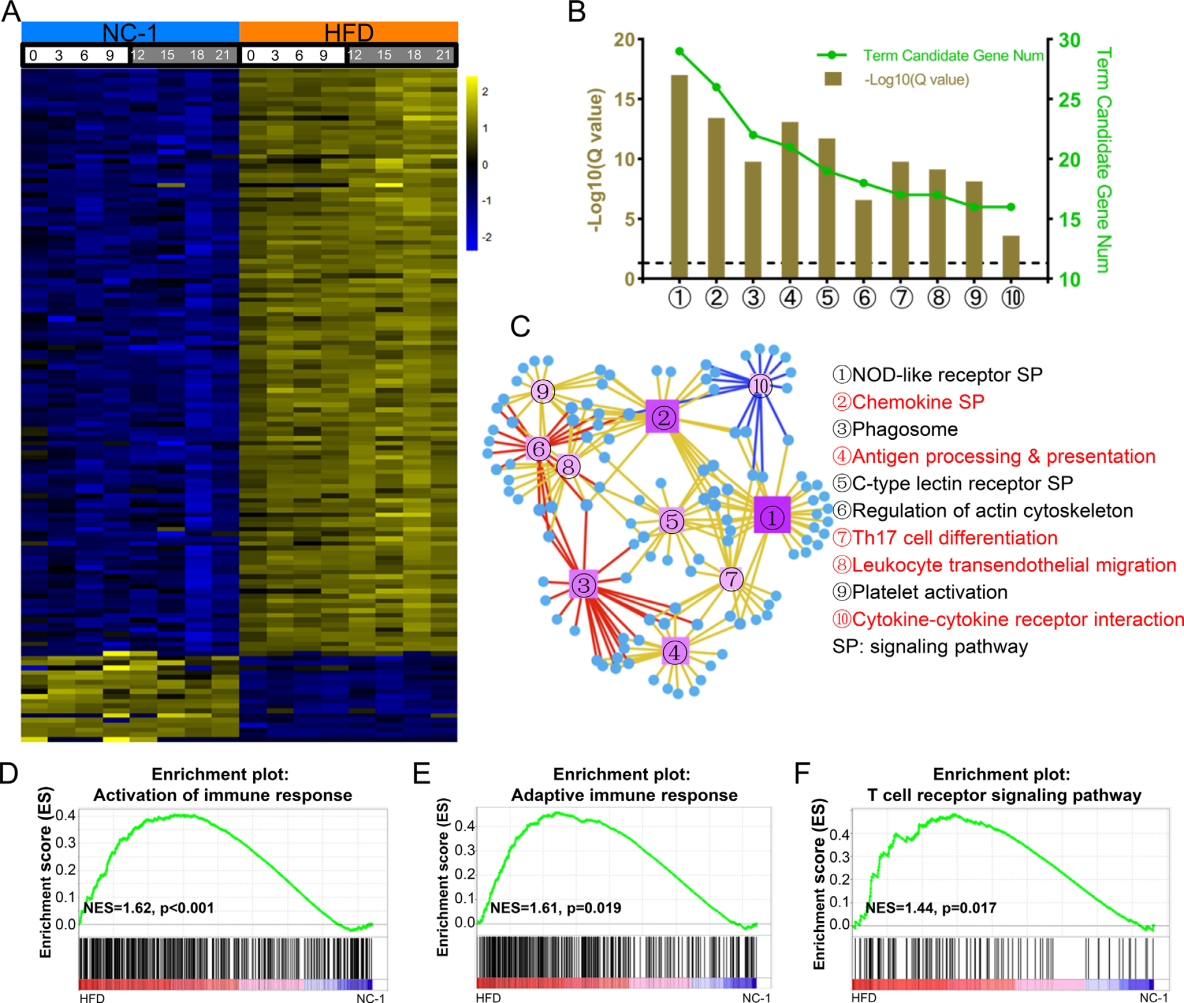
HFD alters immune-related circadian transcriptomic profiles. (**A**) Heatmaps of the differentially expressed immune-related genes (fold change ≥ 2 or ≤ 0.5, BH-adjusted *P* < 0.05). The expression levels of these genes at different times of the day were obtained by RNA-Seq analysis of ELGs. The *color bar* indicates the scale for transcript expression from *blue* to *yellow* across eight time points, with the expression range normalized to ±2. (**B**) Annotation of differentially expressed immune-associated transcripts from the KEGG pathway database (*Q* < 0.05). The top 10 annotated pathways are shown. The *dashed lines* represent *Q* = 0.05. (**C**) KEGG network diagram of differentially expressed immune-associated transcripts. The *circles* represent transcripts. The color and size of the circles indicate the number of transcripts connected to each node. The darker the color and the larger the box, the more transcripts connected to the node. Differently colored lines represent different pathway classifications. The *red*, *blue*, and *yellow lines* represent cellular processes, environmental information processing, and organismal systems, respectively. (**D**–**F**) GSEA results showing enrichment plots for activation of the immune response (**D**), adaptive immune response (**E**), and T cell receptor signaling pathway (**F**), in the ELGs of NC-1 and HFD mice.

To determine the effect of HFD on immune-associated conditions in ELGs, we compared the expression of immune-associated transcripts over a 24-hour cycle in NC- and HFD-fed ELGs. We selected 141 differentially expressed genes (fold change ≥ 2 or ≤ 0.5, BH-adjusted *P* < 0.05), of which 122 were upregulated and 19 were downregulated (Fig. 8A; Supplementary Table S11). The biological functions of these transcripts were annotated using KEGG pathway enrichment analysis. The top 10 enriched pathways (*Q* < 0.05) are shown in Figure 8B and listed in Supplementary Table S12. Figure 8C shows the specific associations of immune-associated differentially expressed genes with specific pathways. To evaluate the changes in immune homeostasis in HFD-fed ELGs, GSEA analysis was performed on the two groups of transcripts. The results showed that the differentially expressed genes were significantly enriched in the signal pathways listed in Figures 8D to 8F and Supplementary Figure S3.

**Figure 9.**
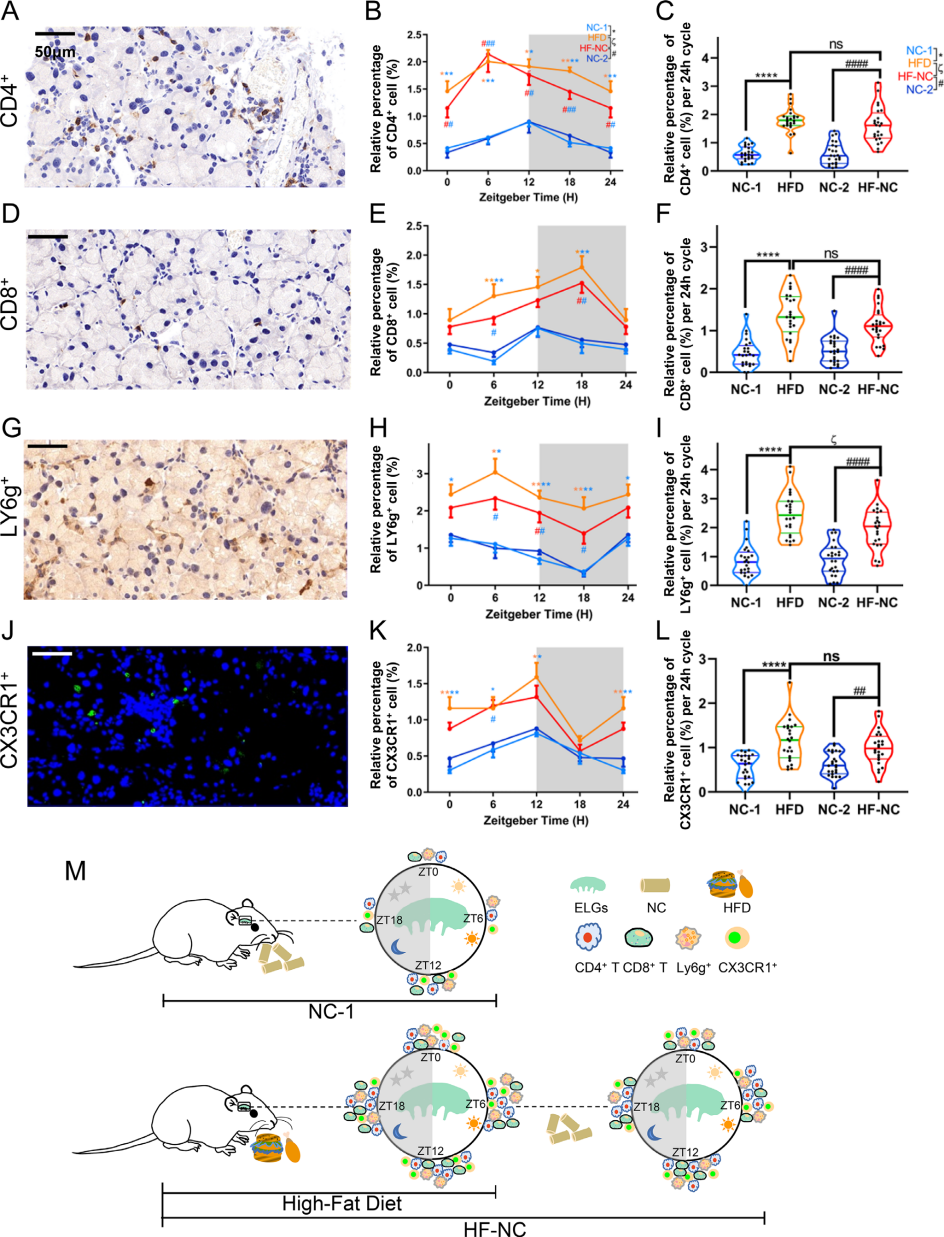
HFD changes the immune homeostasis of lacrimal glands in mice. (**A**, **D**, **G**, **J**) Representative immunohistochemistry images showing CD4^+^ T lymphocytes (**A**), CD8^+^ T lymphocytes (**D**), Ly6g^+^ neutrophils (**G**), and CX3CR1^+^ macrophages (**J**) of ELGs from the HFD group at ZT12. Magnification, 400×. *Scale bar*: 50 µm. (**B**, **E**, **H**, **K**) Diurnal oscillations in the relative abundance of CD4^+^ T lymphocytes (**B**), CD8^+^ T lymphocytes (**E**), Ly6g^+^ neutrophils (**H**), and CX3CR1^+^ macrophages (**K**) in the immunohistochemistry images of ELG sections from NC-1, HFD, NC-2, and HF-NC ELGs at 6-hour intervals; *n* = 6 mice per group per time point (every 6 hours). One-way ANOVA using Bonferroni correction: ^*^significant difference between NC-1 and HFD groups; ^#^significant difference between NC-2 and HF-NC groups; ^ζ^significant difference between HFD and HF-NC groups. *P < 0.05, **P < 0.01, ***P < 0.001, ****P < 0.0001. *Gray shading* indicates dark phase. (**C**, **F**, **I**, **L**) Average relative abundance of CD4^+^ T lymphocytes (**C**), CD8^+^ T lymphocytes (**F**), Ly6g^+^ neutrophils (**I**), and CX3CR1^+^ macrophages (**L**) in the immunohistochemistry images of ELG sections from NC-1, HFD, NC-2, and HF-NC ELGs; *n* = 24 mice per group. ^*^Significant difference between NC-1 and HFD groups; ^#^significant difference between NC-2 and HF-NC groups; ^ζ^significant difference between HFD and HF-NC groups. *P < 0.05, **P < 0.01, ****P < 0.0001; ns, not significant. (**M**) Schematic diagram displaying diurnal changes in the number of different immune cells recruited to lacrimal glands. *Gray shading* indicates dark phase.

In a steady state, different types of immune cells migrate diurnally to lacrimal glands from peripheral circulation.^63^ To understand the effect of HFD feeding on the rhythmic recruitment of different immune cells to lacrimal glands, we used immunohistochemical stain and counted the number of CD4^+^ T cells, CD8^+^ T cells, and Ly6g^+^ neutrophils in lacrimal glands at 6-hour intervals (Figs. 9A–9M). In both NC-1 and HFD groups, the numbers of the different kinds of immune cells recruited to the lacrimal glands showed a significant circadian rhythm (Figs. 9B, 9E, 9H, 9K, 9M). In NC-fed ELGs, the numbers of CD4^+^ T cells, CD8^+^ T cells, and CX3CR1^+^ cells peaked at ZT12, whereas Ly6g^+^ neutrophils peaked at ZT0. However, after HFD-feeding treatment, the peak of CD4^+^ T cells and Ly6g^+^ neutrophils shifted to ZT6, the peak of CD8^+^ T cells shifted to ZT18, and the peak of CX3CR1^+^ macrophages/monocytes did not change (Figs. 9B, 9E, 9H, 9K). Importantly, the number of these different kinds of immune cells, both at different time points and cumulative number from four time points, significantly increased compared with that of NC-fed ELGs (Figs. 9C, 9F, 9I, 9L). Interestingly, in HF-NC ELGs, the pattern and number of immune cell recruitment to the ELGs were comparable to those of HFD-fed ELGs (Figs. 9A–9M). Collectively, these data suggest that HFD altered the pattern and number of normal circadian recruitment of some immune cells to the ELGs and that a short-term feeding reversal regimen did not restore these to the normal status.

### HFD Feeding Alters Secretion Response and Neuro-Associated Gene Expression

**Figure 10.**
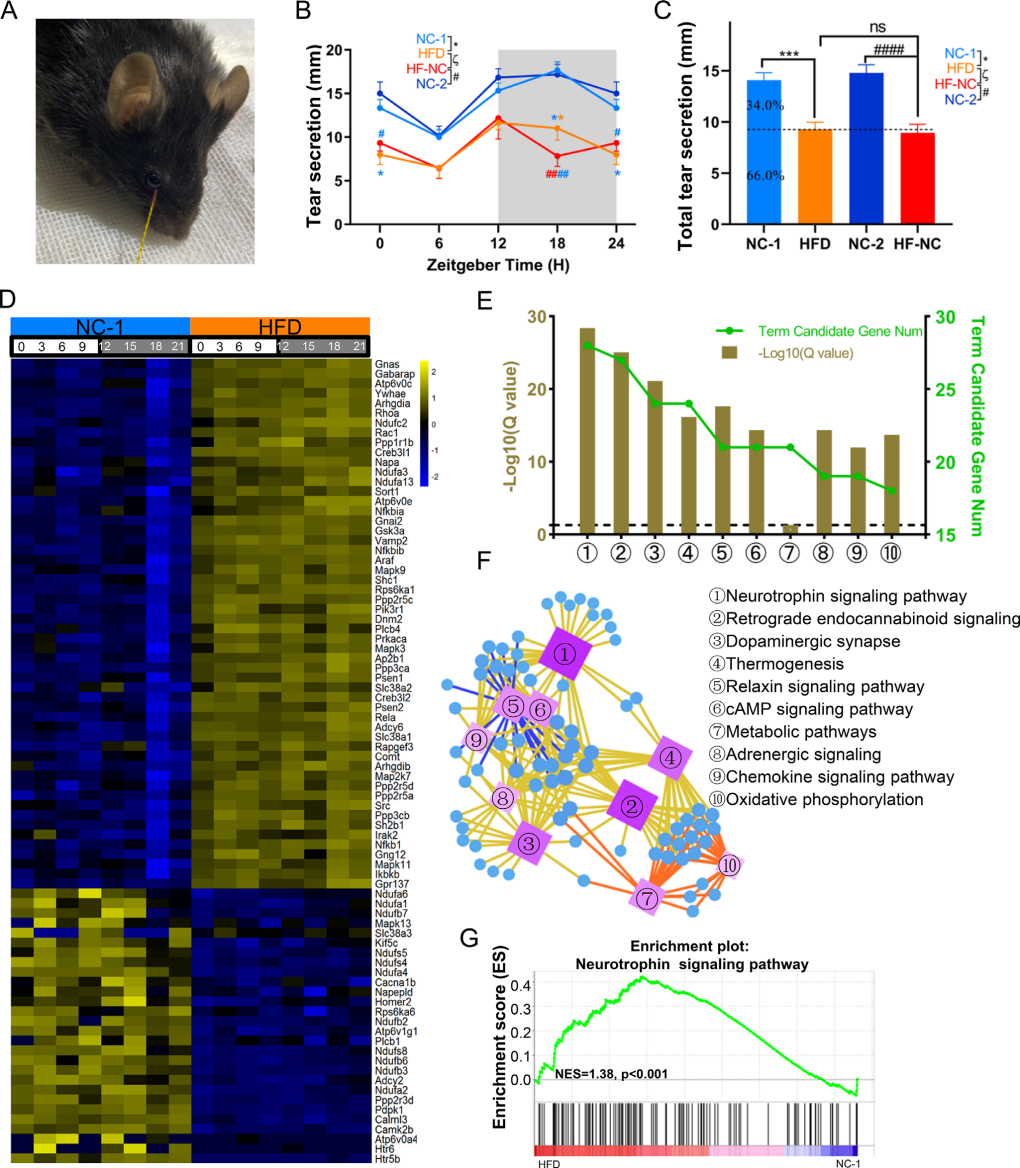
HFD alters the neural-related transcriptome and the secretory functions of murine lacrimal glands. (**A**) Measurement of tear secretion in mice using phenol red threads after pilocarpine stimulation. (**B**) Temporal changes in tear secretions at different ZTs; *n* = 6 mice per group per time point (every 6 hours). One-way ANOVA using Bonferroni correction: ^*^significant difference between NC-1 and HFD groups; ^#^significant difference between NC-2 and HF-NC groups; ^ζ^significant difference between HFD and HF-NC groups. *P < 0.05, **P < 0.01, ****P < 0.0001. *Gray shading* indicates dark phase. (**C**) Total tear secretions in a circadian cycle in NC-1, HFD, NC-2, and HF-NC mice; *n* = 24 mice per group. One-way ANOVA using Bonferroni correction: ^*^significant difference between NC-1 and HFD groups; ^#^significant difference between NC-2 and HF-NC groups; ^ζ^significant difference between HFD and HF-NC groups. ***P < 0.001, ****P < 0.0001; ns, not significant. (**D**) Heatmaps of the differentially expressed neural activity-related genes (fold change ≥ 1.50 or ≤ 0.67, BH-adjusted *P* < 0.05). The expression levels of these genes at different times of the day were obtained by RNA-Seq analysis of ELGs. The *color bar* indicates the scale for transcript expression from *blue* to *yellow* across eight time points, with the expression range normalized to ±2. (**E**) Annotation of differentially expressed neural activity-related genes from the KEGG pathway database (*Q* < 0.05). The top 10 annotated pathways are shown. The *dashed lines* represent *Q* = 0.05. (**F**) KEGG network diagram of differentially expressed neural activity-related transcripts. The *circles* represent transcripts. The color and size of the circles indicate the number of transcripts connected to each node. The darker the color and the larger the box, the more transcripts connected to the node. Differently colored lines represent different pathway classifications. The *yellow*, *orange*, and *blue lines* represent organismal systems, metabolism, and environmental information processing, respectively. (**G**) GSEA results showing the enrichment plots for the neurotrophin signaling pathway in the ELGs of NC-1 and HFD mice.

To determine the effects of HFD on the secretion response of the lacrimal gland, we assayed the secretion response of the ELGs to pilocarpine, a muscarinic cholinergic agonist, using phenol red threads over four different circadian rhythms at 6-hour intervals (Fig. 10A). The tear secretory responses from four animal groups showed rhythmic changes (Fig. 10B). Consistent with our previous findings, the secretion volume peaked at ZT18 and troughed at ZT6 in the NC-fed group.^58^ However, the secretion volume peaked at ZT12 and troughed at ZT6 in the HFD- and HF-NC–fed groups. Moreover, the secretion volume at each time point in the HFD- and HF-NC–fed groups declined compared with that in NC-fed group, and the differences were significant at ZT0 and ZT18 (Fig. 10B). Interestingly, there was no significant difference between the HFD-fed and HF-NC–fed groups (Fig. 10B). The total tear secretion (at five time points) was significantly lower in the HFD- and HF-NC–fed mice than in NC-fed mice (Fig. 10C). Taken together, these data suggest that HFD feeding significantly reduced the secretory response of lacrimal glands, and switching to NC food for 2 weeks did not reverse these changes.

The secretion process of the lacrimal glands is closely controlled by autonomous nerves,^51^^,^^71^ and especially the parasympathetic nerves.^72^ To understand the molecular mechanisms underlying the decrease in tear production after HFD feeding, we selected differentially expressed neuro-related genes between NC-1 and HFD ELGs (fold change ≥ 1.50 or ≤ 0.67, BH-adjusted *P* < 0.05). We found 82 differentially expressed genes, of which 54 were upregulated and 28 were downregulated (Fig. 10D). KEGG enrichment analysis showed that these transcripts mainly were enriched in the neurotrophin/retrograde endocannabinoid/relaxin/cAMP/adrenergic signaling pathway, dopaminergic synapse, and thermogenesis (Figs. 10E–10G; Supplementary Table S13). Collectively, these results indicate that HFD feeding diminished the tear secretory response and altered the neural activities of ELGs.

## Discussion

**Figure 11.**
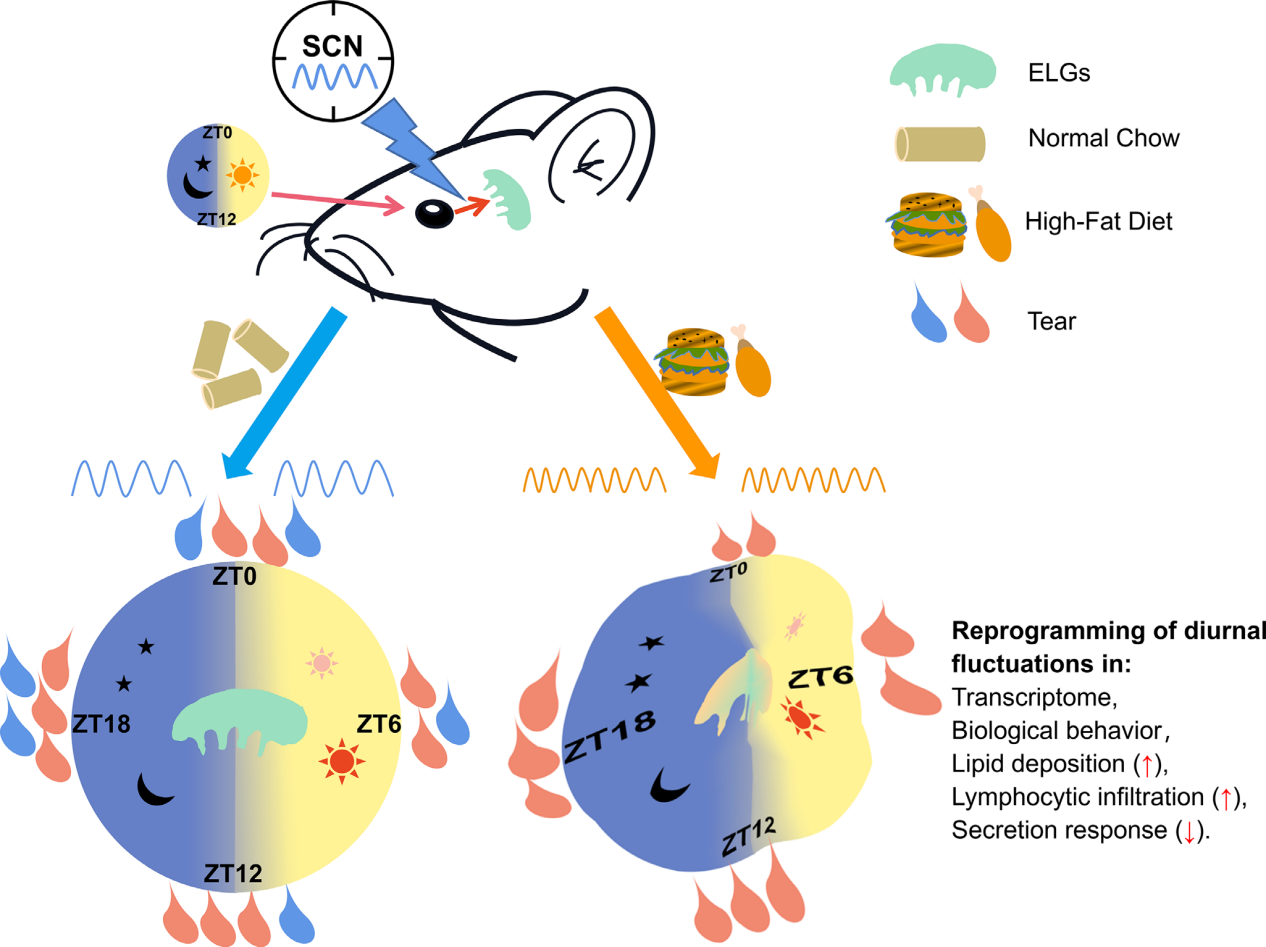
A schematic displaying the effects of an HFD on the circadian rhythm of lacrimal glands. Under a normal light/dark cycle, the nutritional challenge from an HFD leads to the alterations of rhythmic patterns in the lacrimal gland, including changes in the rhythmic transcriptomic profile, lipid deposition in the lacrimal glands, altered rhythmic recruitment of immune cells to the lacrimal glands, and a decrease in the secretion response of lacrimal glands.

To our knowledge, this is the first study to show that high-fat nutritional stress uniquely impacts the circadian rhythmicity of murine ELGs. A 2-week HFD regimen significantly affected the diurnal features of the ELGs, including their cycling transcriptomic profiles, immune cells trafficking to the ELGs, and secretion response to an acetylcholine receptor agonist (Fig. 11). Surprisingly, the distributions of most phase-dependent pathways in the ELGs were reversed from the light phase in NC-fed animals to the dark phase in HFD-fed animals. These data suggest that nutritional challenges from short-term high-fat dietary intake reschedule the circadian rhythms in the ELGs.

In mammals, the circadian rhythm is controlled by the classical retinohypothalamic tract (RHT) and a variety of exogenic zeitgebers,^73^^–^^75^ including nutritional alternations,^46^^,^^69^^,^^76^ diet timing,^40^^,^^77^ temperature,^78^ and sleep.^79^ Disruptions in these factors cause a disorder of the circadian rhythm and promote the occurrence of some diseases, such as metabolic syndrome and type 2 diabetes.^80^^,^^81^ The lacrimal glands are important exocrine glands, and their rhythmic characteristics, such as metabolism, immunity, and secretion, are also affected by the RHT pathway and different exogenous timing factors.^58^^,^^63^ Our previous studies have shown that metabolic stress caused by high fructose intake can significantly change the circadian transcriptomic profile of the lacrimal glands.^57^ Moreover, the metabolic stress caused by streptozotocin-induced hyperglycemia can cause significant rhythmic changes at the transcriptional level and circadian changes in the cell size and secretory response of lacrimal glands.^58^ Consistent with these patterns, a high fat intake—corresponding to a Western dietary model—also caused changes in the circadian rhythm of lacrimal glands in this study. However, these changes were different from those observed in lacrimal glands due to high fructose intake and in other organs (such as the liver) due to HFDs. A characteristic feature of these changes is that the phase of circadian transcriptome expression is reversed from a daytime phase to a nighttime phase in normal mice. Recent studies have shown that the circadian rhythmic activities of different organs are closely linked^82^^,^^83^ and that high fat intake disrupts this link.^82^ Thus, we speculate that an HFD may alter these links between the circadian machineries of other organs (such as the lacrimal glands), leading to a misalignment or reversal in the phase of the circadian transcriptome. However, the mechanism of how the lacrimal gland coordinates and communicates with other important organs (such as the liver) in the normal state and how an HFD affects the aforementioned inter-organ links require further exploration.

The HFD altered the oscillation phase of 79.85% of the shared rhythmic genes in the lacrimal glands in our study. This was similar to the effect of an HFD on liver transcriptomic profiles, which caused a phase shift in 66% of shared rhythmic genes.^44^ The difference was that, after HFD feeding, 79% of the shifted rhythmic genes in the HFD-fed liver shifted forward in phase,^44^ whereas 64.32% of those in the HFD-fed lacrimal glands shifted backward in phase. This suggests that HFD affects circadian transcriptomic profiles in a tissue-specific manner. The oscillation characteristics of diurnally rhythmic genes and their KEGG functional annotations were also significantly changed between the two groups. Although the time-series analysis revealed four oscillatory modes in the transcriptome over a 24-hour cycle in both groups, the KEGG analysis of expressed genes in each oscillatory mode showed differentially enriched pathways and annotations. These findings show that HFD feeding changes the rhythmic transcriptome—and the related functions—of lacrimal glands.

The maintenance of lacrimal gland secretory functions requires a normal metabolic process under neural control.^51^ In this study, we found that HFD feeding dramatically altered the neural activity- and metabolism-associated transcriptomic profiles of murine ELGs over a 24-hour cycle. The former finding indicates the alteration of synapse-related pathways, whereas the latter indicates the activation of several lipid metabolism-related signaling pathways. Consistent with this, the histological analysis revealed that lipid accumulation in the lacrimal glands was higher in HFD-fed mice than in NC-fed mice. We also found that the secretory response of lacrimal glands (stimulated by a cholinergic agonist) decreased significantly in HFD-fed animals over a 24-hour cycle.

Lacrimal glands harbor different types of immune cells.^84^^,^^85^ As in other peripheral organs/tissues,^86^^–^^89^ immune cells are rhythmically trafficked from the circulation to lacrimal glands.^63^ In a previous study, we found that this circadian trafficking was affected by a light cure in a mouse model of jet lag.^63^ Several recent studies have shown that HFD significantly alters the rhythms of physiological function in the liver and brain.^20^^,^^44^^,^^46^^,^^69^^,^^82^ Here, we show that HFD-induced metabolic stress also causes significant alterations in the immune-related transcriptome of lacrimal gland tissue. Moreover, an HFD changes the circadian phase and amplitude of trafficking of a variety of immune cells (mainly CD4^+^ and CD8^+^ T cells and neutrophils) to the lacrimal glands over a 24-hour cycle. The higher expression of immune-related genes and the increased recruitment of immune cells in HFD-treated lacrimal glands indicate a sub-inflammatory condition. This is consistent with recent findings showing that a Western diet can activate innate immune cells to evoke a state of lasting metabolic inflammation.^90^^–^^92^ Therefore, exploring efficient preventive and therapeutic strategies is of great importance.

Reversing the negative effects of HFD on lacrimal gland functions depends on the reversibility of the pathological changes. Our results showed that after diet reversal intervention, the general parameters (fluid intake, pellet intake, and plasma glucose level) and the level of lipid deposition in lacrimal glands returned to nearly NC-fed levels. However, important factors of circadian behavior—including locomotor activities, core body temperature, immune cell recruitment to the ELGs, and the secretory response of ELGs—did not reverse to normal levels. These results suggest that the aforementioned HFD-induced pathological alterations in ELGs may persist for longer periods of diet reversal. This may also be because the neural regulatory circuit had not been restored.^51^^,^^72^ Thus, it would be interesting to determine whether this diurnal regulation remains permanently disrupted.

As for the potential clinical relevance of this study, first, it is well known that a high-fat diet is associated with the development of type 2 diabetes mellitus.^93^^,^^94^ Limited clinical evidence shows that patients with type 2 diabetes mellitus present with lower tear volumes and a higher incidence of lacrimal gland hypofunction than age-matched controls.^95^^–^^97^ In addition, a high-fat diet is thought to be an important contributor to the global obesity epidemic.^98^^,^^99^ Metabolic stress in patients with metabolic syndrome is accompanied by circadian dysfunction due to disturbance of clock-related genes in several key organs.^100^^–^^102^ Consistent with these studies, the data from the present study suggest that high levels of dietary fat may also contribute to change of lacrimal gland structure and dysfunction of secretion by altering the circadian rhythm of the human lacrimal gland.^103^^,^^104^ However, a large number of studies are still needed to explore the clinical relevance.

Although this study revealed that a short-term HFD can significantly rewire the circadian rhythm of mouse ELGs, it had some limitations. First, this study only observed the effects of a short-term HFD on the circadian rhythms of the transcriptome, metabolism, immunity, and secretory functions in lacrimal glands. The effects of a long-term Western diet (HFD with high sugar intake) and of HFD-induced obesity and metabolic syndrome on lacrimal gland functions are unclear and should be explored. Second, this study only observed the alterations in the transcriptome, structure, and secretory functions of the lacrimal gland; however, analyzing metabolomic and proteomic data can provide more insightful information. Third, this study used only male animals as a study model. Like other physiological systems or organs,^105^^–^^107^ the physiological functions of the lacrimal gland and its response to internal and external stresses are also sexually dimorphic.^108^^–^^110^ More importantly, a recent study demonstrated that there is sexual dimorphism in body clocks.^111^ Therefore, exploring the mechanisms associated with female sex and differences between the sexes might provide important sex-specific data in the future. Fourth, although the transcriptomic analysis in this study provides many potential target molecules and signaling pathways, the corresponding interventions and therapeutic measures have not been explored. Fifth, consistent with other studies,^112^^,^^113^ HFD maintenance reduces the amount of fluid intake. Whether this alteration in water intake behavior associated with HFD interferes with the circadian rhythmicity of the lacrimal gland and its respective weight deserve further exploration, particularly with respect to the rhythmicity of lacrimal gland secretion. Finally, our circadian transcriptomic data were obtained from the bulk RNA of whole lacrimal glands. However, uncovering the changes in the circadian transcriptome of different cell populations in the ELGs will help elucidate the corresponding molecular mechanisms. These can be analyzed in future studies using the recently developed single nucleus sequencing and single-cell RNA-sequencing technologies.^114^^,^^115^

In conclusion, our study reveals that the nutritional challenge from an HFD modulates an unexpected plasticity of the lacrimal glands in terms of its circadian transcriptomic profile and diurnal changes in cell size, immune cell recruitment, and secretion response. Our results highlight future avenues to explore how nutritional components from different diets affect the daily oscillations of some physiological functions by altering specific molecular events in the lacrimal glands.

## Supplementary Material

Supplement 1

Supplement 2

Supplement 3

Supplement 4

Supplement 5

Supplement 6

Supplement 7

Supplement 8

Supplement 9

Supplement 10

Supplement 11

Supplement 12

Supplement 13

Supplement 14

Supplement 15

Supplement 16
