# Supplementary material for: High-Fat Nutritional Challenge Reshapes Circadian Signatures in Murine Extraorbital Lacrimal Glands
Source: Invest Ophthalmol Vis Sci. 2022 May 19;63(5):23. doi: 10.1167/iovs.63.5.23 (PMC9123521; doi:10.1167/iovs.63.5.23)
Supplement: Supplement 1 [file iovs-63-5-23_s001.pdf]

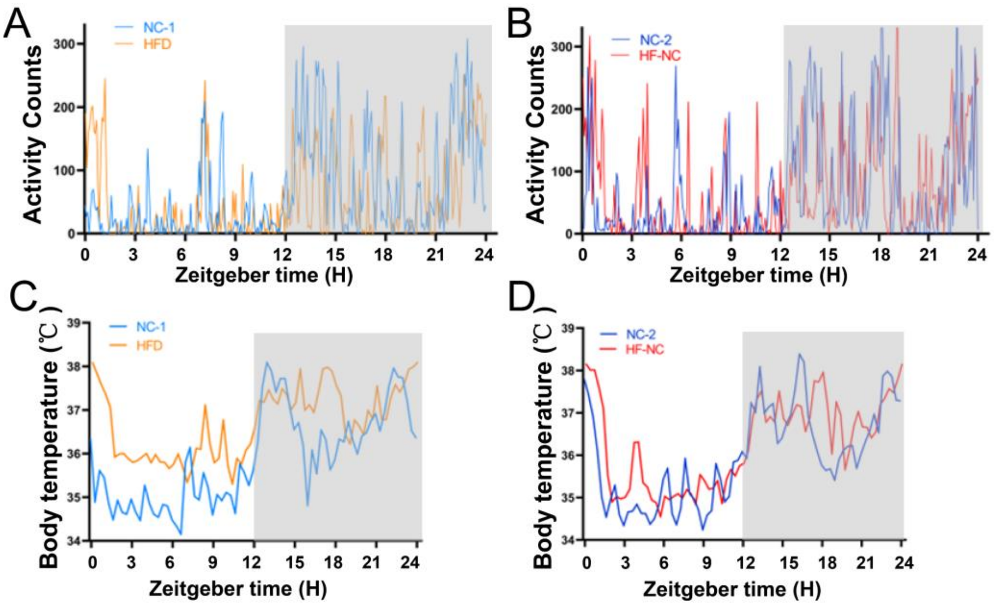

FIGURE S1. High-fat diet feeding affects animal behavior

(A,B) Representative rhythmic characteristics of locomotor activity in animals in NC-1 and HFD groups **(A)**, and NC-2 and HF-NC groups **(B)** over the last 24-h light cycle (12 h light/12 h dark) during the dietary intervention. Gray shading: dark phase.

(C,D) Representative rhythmic characteristics of core body temperature in animals in NC-1 and HFD groups **(C)**, and NC-2 and HF-NC groups **(D)** over the last 24-h light cycle (12 h light/12 h dark) during the dietary intervention. Gray shading: dark phase.
