# Supplementary material for: High-Fat Nutritional Challenge Reshapes Circadian Signatures in Murine Extraorbital Lacrimal Glands
Source: Invest Ophthalmol Vis Sci. 2022 May 19;63(5):23. doi: 10.1167/iovs.63.5.23 (PMC9123521; doi:10.1167/iovs.63.5.23)
Supplement: Supplement 2 [file iovs-63-5-23_s002.pdf]

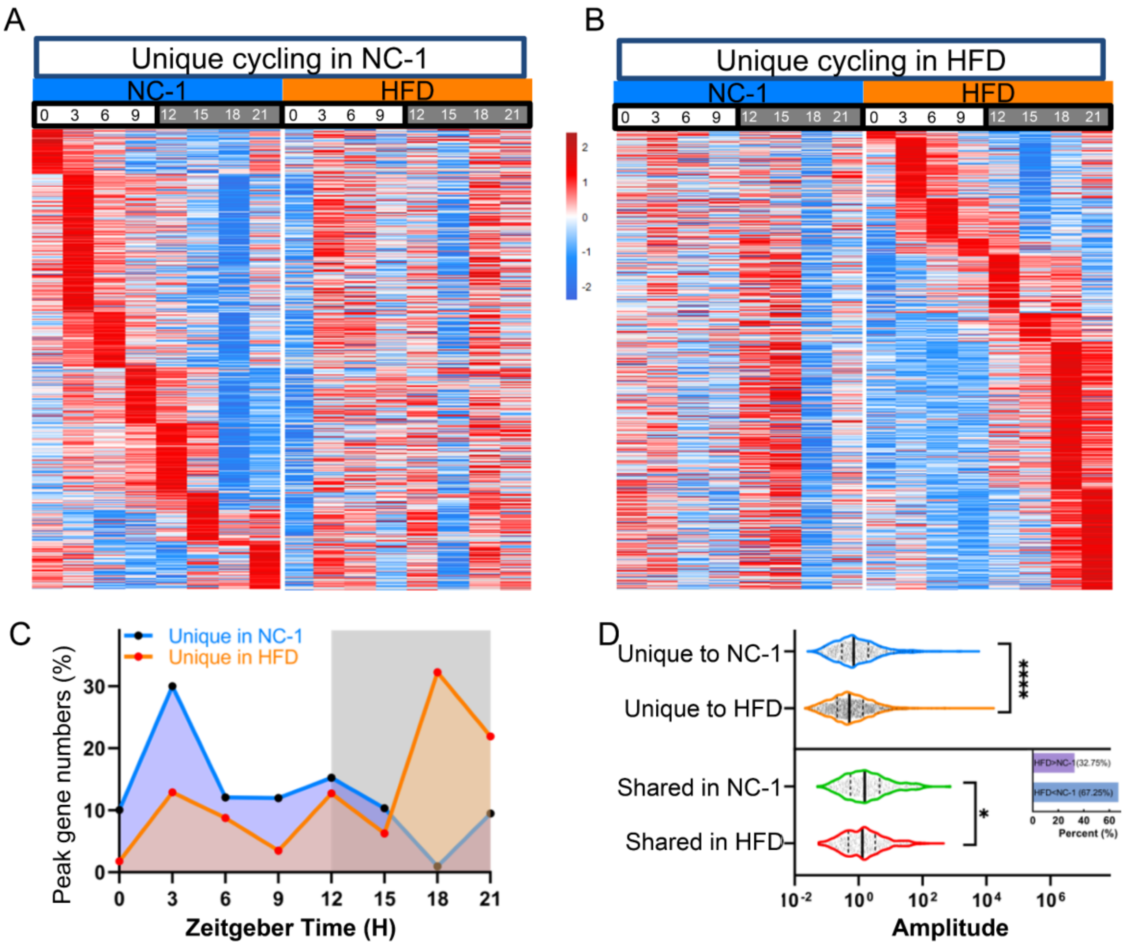

FIGURE S2. HFD alters the characteristics of the circadian transcriptome

(A) Heatmaps showing the expression levels of 1037 oscillating genes unique to the NC-1 group at different ZT time points (JTK algorithm, adjusted $P<0.05$ and expression≥0.1). The expression changes for the NC-1 (*left*) and HFD (*right*) groups are shown for 8 time points across the 24-h cycle. The colored bar indicates the degree of gene expression at 8 time points. The color bar indicates the scale for transcript expression from blue to red across eight time points, with the expression range normalized to ±2.

(B) Heatmaps showing the expression levels of 2147 transcripts unique to the HFD group at different ZT time points (JTK algorithm, adjusted $P<0.05$ and expression ≥0.1). The expression changes for the HFD (*right*) and NC-1 (*left*) groups are shown for the 8 time points across the 24 h cycle. The color bar

indicates the scale for transcript expression from blue to red across eight time points, with the expression range normalized to ±2.

(C) The distribution of peak gene expressions in NC-1 and HFD ELGs over different ZT time points. Gray shading: dark phase.

(D) Oscillation amplitudes of unique cycling genes in the ELGs of NC-1 and HFD mice (*up*). Amplitudes of shared cycling genes in the ELGs of NC-1 and HFD mice (*down*). Nonparametric test (Mann-Whitney test): *$P<0.05$, ****$P<0.0001$.
