# Supplementary material for: High-Fat Nutritional Challenge Reshapes Circadian Signatures in Murine Extraorbital Lacrimal Glands
Source: Invest Ophthalmol Vis Sci. 2022 May 19;63(5):23. doi: 10.1167/iovs.63.5.23 (PMC9123521; doi:10.1167/iovs.63.5.23)
Supplement: Supplement 3 [file iovs-63-5-23_s003.pdf]

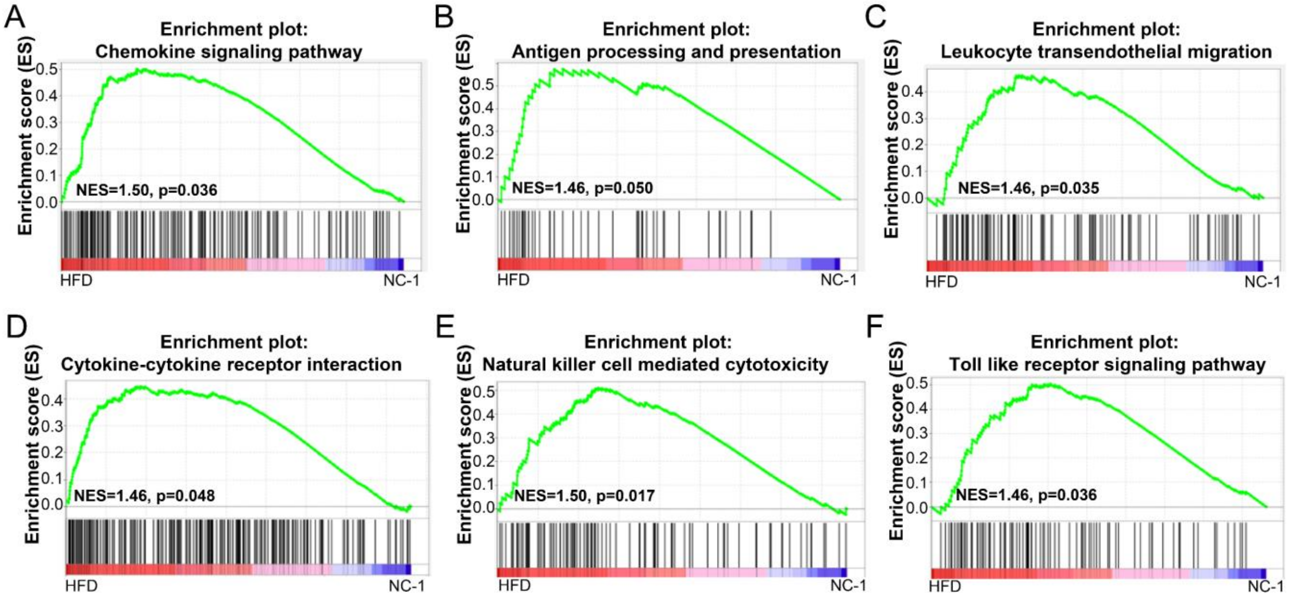

FIGURE S3. GSEA results showing the enrichment plots for the chemokine signaling pathway **(A)**, antigen processing and presentation **(B)**, leukocyte transendothelial migration **(C)**, cytokine-cytokine receptor interaction **(D)**, natural killer cell-mediated cytotoxicity **(E)**, and Toll-like receptor signaling pathway **(F)** in the ELGs of NC- and HFD-fed mice. NES: normalized enrichment score.
