# Supplementary material for: High-Fat Nutritional Challenge Reshapes Circadian Signatures in Murine Extraorbital Lacrimal Glands
Source: Invest Ophthalmol Vis Sci. 2022 May 19;63(5):23. doi: 10.1167/iovs.63.5.23 (PMC9123521; doi:10.1167/iovs.63.5.23)
Supplement: Supplement 4 [file iovs-63-5-23_s004.pdf]

Supplemental Table S1. Composition of diets.

	Normal chow	High fat diet
**Energy (kcal%)**		
Protein	15	14
Fat	9	60
Carbohydrate	76	26
Total	100	100
**Ingredient (g/kg)**		
Casein	140	195
Dextrin	155	225
Sucrose	100	89
Soybean Oil	40	33
Lard	0	301
Cellulose	50	69
AIN93 Mineral Mix	35	68
AIN93 Vitamin Mix	10	14
L-Cystine	1.8	3
Choline Bitartrate	2.5	3
Corn Starch	466	0
tert-Butylhydroquinone	0.008	0.067
Total	1000	1000
